# Synthesis of salicylic acid phenylethyl ester (SAPE) and its implication in immunomodulatory and anticancer roles

**DOI:** 10.1038/s41598-022-12524-7

**Published:** 2022-05-24

**Authors:** Arup Jyoti Das, Monoj Kumar Das, Salam Pradeep Singh, Partha Pratim Saikia, Neelu Singh, Johirul Islam, Aftab Ansari, Pronobesh Chattopadhyay, Paulraj Rajamani, Tatsuro Miyaji, Sankar Chandra Deka

**Affiliations:** 1 Department of Food Engineering and Technology, Tezpur University, Tezpur, Assam India; 2 Department of Molecular Biology and Biotechnology, Tezpur University, Tezpur, Assam India; 3 Department of Chemistry, N.N. Saikia College, Titabar, Assam India; 4 School of Environmental Sciences, Jawaharlal Nehru University, New Delhi, India; 5 Division of Pharmaceutical Technology, Defence Research Laboratory, Tezpur, Assam, India; 6 Department of Physics, Tezpur University, Tezpur, Assam India; 7 Department of Materials and Life Science, Shizuoka Institute of Science and Technology, Fukuroi, Japan

**Keywords:** Cancer, Cell biology, Computational biology and bioinformatics, Drug discovery, Immunology

## Abstract

Salicylic acid phenylethyl ester (SAPE) was synthesized by Zn(OTf)_2_-catalyzed selective esterification of salicylic acid and phenylethyl alcohol and studied for its role as an immunomodulatory and anticancer agent. Low toxicity and favorable physical, Lipinski-type, and solubility properties were elucidated by ADME-tox studies. Molecular docking of SAPE against COX-2 revealed favorable MolDockscore, rerank score, interaction energy, internal pose energy, and hydrogen bonding as compared to ibuprofen and indomethacin. An average RMSD of ~ 0.13 nm for the docked complex with stable dynamic equilibrium condition was noted during the 20 ns MD simulation. A low band gap predicting a strong binding affinity at the enzyme’s active site was further predicted by DFT analysis. The ester caused a reduction in the percentage of erythrocyte hemolysis and was shown to be non-cytotoxic against human lymphocytes, CaCo-2, and HepG-2 cells by the MTT assay. Moreover, it’s in vitro efficacy in inhibiting COX-2 enzyme under both LPS stimulated intestinal cells and direct sequestration assays was found to be higher than salicylic acid and indomethacin. The anticancer activity of SAPE was tested on the breast cancer cell line MCF-7, and potential efficacy was exhibited in terms of decreased cell viability. Flow cytometry analysis exhibited the arrest of the cell cycle at G1/G0 and S phases, during which induction of autophagic vesicle formation and decrease in mitochondrial membrane potential was observed owing to increased ROS production. Furthermore, at these phases, the onset of apoptosis along with DNA damage was also observed. Pre-treatment with SAPE in colitis-induced Wistar rats displayed low disease activity index and reduction in the extent of intestinal tissue disruption and lipid peroxidation. A marked increase of anti-oxidative enzymes viz., catalase, GGT, and GST, and a decrease of pro-inflammatory cytokines IL-6 and TNF-α in the intestinal tissue extracts of the treated groups was noted. The results of this study have sufficient credence to support that the synthesised ester (SAPE) be considered as an anti-oxidative and anti-inflammatory compound with therapeutic potential for the effective management of cancer.

## Introduction

Phenolic esters are partially soluble in water and exhibits antioxidant properties and found abundantly in natural products^1^. They possess a variety of pharmacological activities like anti-inflammatory, antioxidant, immunomodulatory, and anti-cancer effects, e.g., caffeic acid phenethylester (CAPE), a phenolic active component of honey bee propolis^2^. These compounds are unsophisticated structurally; however, their synthesis is typically complicated due to the burden of protecting groups for improved chemoselectivity, or the use of harsh conditions or excessive use of one of the reactants^3,4^. Esterification yields are linked to the electronic distribution of phenolic acids which affect the reactivity of the carboxylic function and also the carbon chain length of the alcohols^1^. Esterification of the carboxylic acid function evinced higher anti-inflammatory activities with reduced ulcerogenic effect when compared with standard compounds^5^. Moreover, esterification of acetylsalicylic acid, salicylic acid, flufenamic acid, tolmetin and various other non steroidal anti inflammatory acids result in methyl esters production with lower gastric ulcerogenic activity^6^.

Salicylic acid is a pharmacologically active phenolic compound and is found in various plants. Its mode of action includes prostaglandin synthesis inhibition and suppression of transcription of genes for cyclooxygenase^7^. Anti-cardio cerebral vascular diseases and anti-cancer activities of salicylic acid are being reported^8^. Acetylsalicylic acid which is a derivative of salicylic acid is one of the world’s most widely used anti-inflammatory medicines and induces anti-cancer activity^57^. Rigas and Kozoni^10^ synthesized a new phenylester derivative of aspirin and this compound inhibited the growth of human colon adenocarcinoma cells (HT-29) through a combination of antiproliferative and proapoptotic effects. Çalışkan et al.^11^ also synthesized a series of novel 1-benzyl-5(3)-p-tolyl-1H-pyrazole-3(5)-carboxylic acid derivatives and found that some of these compounds exhibited analgesic and anti-inflammatory activities. Recently, Liu et al.^12^ synthesized a new derivative by the reaction of salicylic acid with α-aminophosphonate and demonstrated that the synthesized salicylic acid derivative had inhibitory activity against liver and cervical cancer cell lines.

Salicylic acid is very sensitive to oxidation, as its hydroxylation by liver microsomal hydroxylases forms gentisic acid, which can react further to produce other hydroxylated derivatives by HO attack. However, oxidized salicylate derivatives possess increased antioxidant activity, partly due to their capacity to function as single electron or hydrogen atom donors^13^. These phenolic derivatives have low acute toxicity and the second OH group at either ortho or para position increases the antioxidant activity. Moreover, the activity of monophenols increases considerably with one or two methoxylic substituents^14^. The salicylic acid esters have a broad spectrum of biological activities, and the esterfication of salicylic acid with methanol have been carried out using different catalysts like Ce^4+^ modified cation-exchange resins^15^ and anion modified metal oxides like zirconia, alumina and silica^16^.

Our previous studies with rice beer (a traditional fermented product of Assam, India), revealed the presence of a considerable amount of salicylic acid in the starter plants^17^ and 2-phenylethanol (PEA) in the final product^18^. PEA is an aromatic alcohol found in fermented foods and various yeast strains produce it from L-phenylalanine via the Ehrlich pathway involving three enzymes^19^. Since no reports were available on the esterification of salicylic acid with phenylethyl alcohol using zinc trifluoromethanesulfonate [Zn(OTf)_2_] as a catalyst, hence for the first time salicylic acid phenylethyl ester (SAPE) was synthesized by Zn(OTf)_2_-catalyzed selective esterification. The synthesized ester was further tested for its immunomodulatory role as an antioxidative, anti-inflammatory and anti-cancer agent via in silico, in vitro, and in vivo approaches.

## Results and discussions

### Structural validation of synthesised SAPE

The reaction for the production of SAPE is illustrated in Fig. SF1. The 40:60 ethyl acetate:hexane mixture yielded the best results in terms of compound concentration. ^13^C NMR and ^1^H NMR plots of the synthesized SAPE molecule are shown in Figs. SF2 and SF3, respectively. FTIR plot of the synthesized SAPE molecule is shown in Fig. SF4. The data obtained are as follows: ^13^C (ppm): 3409, 65.8, 112.1, 118.1, 119.2, 126.12, 129.0, 130.1, 131.1, 135.2, 138.1, 161.8, 170.1. FTIR (cm^−1^): 501, 672, 750, 1070, 1110, 1220, 1292, 1480, 1683, 3150. ^1^H (ppm): 3.1 (t,2H), 4.52 (t,2H), 6.7–6.98 (m,2H), 7.1–7.48 (m,5H), 7.7–7.8 (m,2H), 10.7 (s,1H).

### In silico studies of SAPE

#### ADME properties

The ADME- toxicity properties are directly related to the biological effect of drugs and their fate in an organism, and in silico methods can predict these properties at an early stage in drug design^20^. Solubility, volume of distribution, absorption, blood brain barrier transport, bioavailability, health effects and LD_50_ values of SAPE molecule as predicted by ACD/Labs I-Lab 2.0 are presented in Table ST1. The solubility (LogSw) was found to be − 4.03, while absorption (LogP) was 4.15, blood brain barrier transport (LogP) was 4.15 and volume of distribution was 0.44 L/kg. The maximum passive absorption was 100% from trancellular route, while permeability in human jejunum was 7.51 × 10^−4^ cms^−1^. The probability that the compound has bioavailability of %F (oral) > 30% was 0.811and bioavailability of % F (oral) > 70% was 0.358, whereas, the probability of effect on blood was 0.39; cardiovascular system 0.5; gastrointestinal system 0.6, kidney 0.26, liver 0.14 and lungs was 0.19. The LD_50_ values for rat were found to be 570 mg/kg (administered intraperitoneally) and 2500 mg/kg (administered orally). The CNS activity of SAPE is also shown in Fig. SF5. The predicted physicochemical properties of SAPE viz., molar refractivity, molar volume, parachor, index of refraction, surface tension, density and polarizability and mass spectrometry related properties of SAPE are shown in Table ST4. Moreover, the Lipinski-type properties were also deduced and were found to be favourable (Table ST2).

#### Molecular docking against COX-2

**Figure 1:**
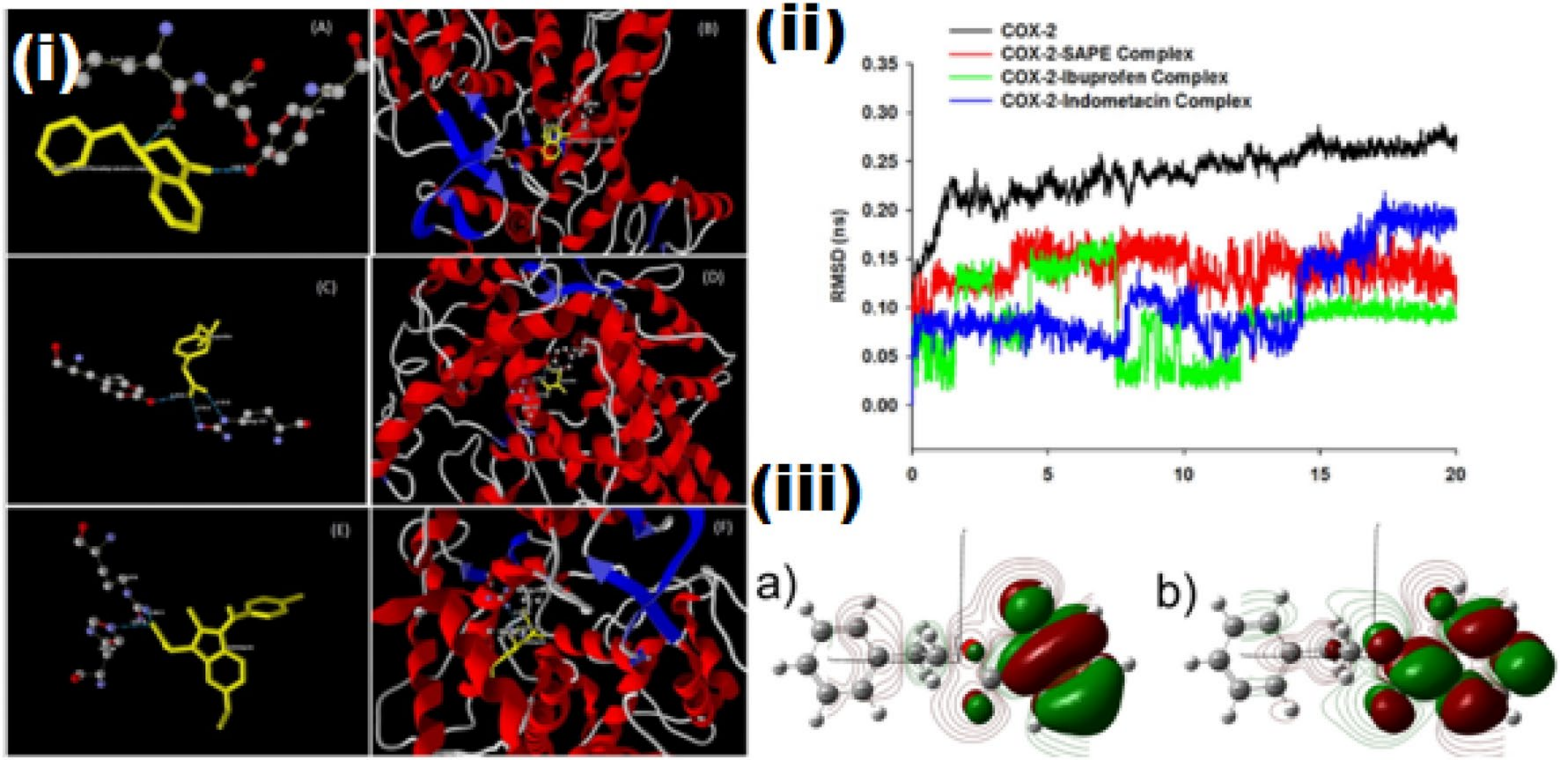
[i] (**A**) Protein–ligand interactions between SAPE and active site residues of COX-2 enzyme (**B**) Binding mode of SAPE at the active site residues of COX-2 enzyme (**C**) Protein–ligand interactions between ibuprofen and active site residues of COX-2 enzyme (**D**) Binding mode of ibuprofen at the active site residues of COX-2 enzyme (**E**) Protein–ligand interactions between indomethacin and active site residues of COX-2 enzyme (**F**) Binding mode of indomethacin at the active site residues of COX-2 enzyme. [ii] MD simulation showing the RMSD plot of the docked protein ligand complex showing that SAPE-COX-2 is more stable than the ibuprofen and indomethacin complex. [iii] (**a**) Molecular orbital depicting the HOMO (E = -6.29 eV) of SAPE calculated at DFT/B3LYP/6-31G level of theory. (**b**) Molecular orbital depicting the LUMO (E = -1.09 eV) of SAPE calculated at DFT/B3LYP/6-31G level of theory.

Initially, the prediction of the potential ligand binding site (active site) on COX-2 displayed the cavity volume to be 56.32 Å^3^, cavity surface of 175.36 Å^2^ and positioned at X: 13.44; Y: 24.14; Z: 24.58 with a binding site radius of 15 Å (Fig. SF6), and molecular docking simulation was performed against SAPE, ibuprofen and indomethacin. The grid-based scoring function, MolDock Score [GRID] was used to evaluate the docking solutions. Since, the compounds under the study possessed several internal degrees of freedom, MolDock SE was used as an alternative search algorithm^21^. The top poses which docked at the active site of COX-2 are shown in Fig. 1[i] A ,B,C,D,E and F, and the values were ranked based on the Rerank score (Table 1).

**Table 1:** Docking scores of the compounds docked against COX-2.

Name	MolDockScore^a^	Rerank Score^b^	Interaction^c^	Internal^d^	H-Bond^e^	MW	LE1f.	LE3^g^
SAPE	− 109.09	− 94.57	− 134.89	25.80	− 2.26	240.30	− 6.06	− 5.25
Ibuprofen	− 92.62	− 77.50	− 100.73	8.10	− 4.33	205.27	− 6.17	− 5.17
Indomethacin	− 102.12	− 84.89	− 132.26	15.11	− 5	357.78	− 4.71	− 3.39

^a^E_Score_ = E_inter_ + E_intra_, where E_inter_ is the ligand–protein interaction energy and E_intra_ is the internal energy of the ligand.

^b^The rerank score is a linear combination of E-inter between the ligand and the protein, and E-intra of the ligand weighted by pre-defined coefficients (more negative means more stable).

^c^The total interaction energy between the pose and the protein (kJ mol^−1^ ) (more negative means more stable).

^d^The internal energy of the pose (the lesser the better).

^e^Hydrogen bonding energy (kJ mol^−1^) (more negative means more stable).

^f^Ligand Efficiency 1: MolDock Score divided by Heavy Atoms count (more negative means more stable).

^g^Ligand Efficiency 3: Rerank Score divided by Heavy Atoms count (more negative means more stable).

The molecular docking scores (Table 1) revealed that SAPE could be docked at the active site of the COX-2 protein with the favourable docking scores; which is an indication that the enzyme usually favours SAPE compared to ibuprofen and indomethacin. In the molecular docking engine, SAPE, ibuprofen, indomethacin were ranked as the top five docking hits based on the MolDock score, Rerank score and interaction energy. The MolDock score employed in the present investigation is derived from the PLP scoring functions originally proposed by Gehlhaar et al.^22^, and later extended by Yang et al.^23^. The docking scoring function, E_score_, is defined as: E_Score_ = E_inter_ + E_intra_, where, E_inter_ is the ligand–protein interaction energy and E_intra_ is the internal energy of the ligand. While, the Rerank score is a linear combination of E-inter (steric, Van der Waals, hydrogen bonding and electrostatic) between the ligand and the protein, and E-intra (torsion, sp2–sp2, hydrogen bonding, Van der Waals and electrostatic) of the ligand weighted by the pre-defined coefficients.

In order to examine the molecular interaction, the ligand–protein interaction analysis for the docking hits SAPE, ibuprofen and indomethacin using the MVD Ligand Energy Inspector were assessed, and Table 2 shows the details of molecular interactions of the docking hits. The ligand–protein interaction including the residue interaction and their interaction distances were determined, whereby SAPE showed molecular interaction with Leu353(O) and Tyr356(OH) (Fig. 1A and B), Ibuprofen with Arg121(NH2) and Tyr356(OH) (Fig. 1C and D) while indomethacin with Arg514(NH) and His90(NE) (Fig. 1E and F). The energy map and electrostatic interaction of the docking hits are shown in Figs. SF7A and B, SF8A and B and Fig. SF9A and B. The energy map of the enzyme contributing towards the steric interaction favourable (green colour), hydrogen acceptor favourable (turquoise colour) and hydrogen donor favourable (yellow colour) and electrostatic interaction of the docked compounds also support the favourable molecular interaction.

**Table 2:** Molecular interaction analysis of the docked ligands with COX-2 enzyme.

Compound	Interaction	Interaction energy	Interaction distance (Å)
SAPE	Leu353(O)–O(11)	− 1.7	2.51
	Tyr3556(OH)–O(16)	− 2.5	3.02
Ibuprofen	Arg121(NE)–O(14)	− 2.5	3.10
	Arg121(NH2)–O(13)	− 1.8	2.75
	Tyr3556(OH)–O(13)	− 2.5	3.10
Indomethacin	Arg514(NH)–O(3)	− 2.5	2.94
	His90(NE)–O(3)	− 2.5	2.87

#### MD simulation of protein–ligand docked complex

The MD simulation was conducted only for the docking hit (SAPE-COX-2, ibuprofen-COX-2 and indomethacin-COX-2 docked complexes). RMSD data for the protein ligand docked complexes were plotted and presented in Fig. 1[ii]. The RMSD backbone of 20 ns MD simulations for understanding the conformational changes of the protein–ligand binding complex and the protein occurring in the dynamic environment were worked out. The RMSD plot clearly explains the variations of the COX-2 protein and the protein–ligand binding complexes. The average RMSD showed ~ 0.13 nm for the SAPE-COX-2 docked complex. While the protein showed an average RMSD deviation of ~ 0.23 nm revealing more stable dynamic equilibrium condition of the protein–ligand complex. This confirms the conformational stability of the docked and the simulated complex during the 20 ns simulation run.

#### DFT study

The HOMO and LUMO energies of SAPE are illustrated in Fig. 1[iii] A and B, respectively. These energies aid in understanding the band gap energy of the docked pose. A low band energy gap indicates a higher reactivity and thus the compound is believed to be strong enough to bind at the protein’s active site. The band gap energy (ΔE_*LUMO−HOMO*_) was − 5.12 eV which confirms a low band gap and will definitely have a strong binding affinity at the active site of the target enzyme. The contour map for depicting the electrostatic potential of SAPE and the predicted IR spectrum of SAPE were also calculated at DFT/B3LYP/6-31G level of theory and these are depicted in Supplementary Fig. SF10 and SF11, respectively.

### In vitro cytotoxicity assay of SAPE in animal cell line models

**Figure 2:**
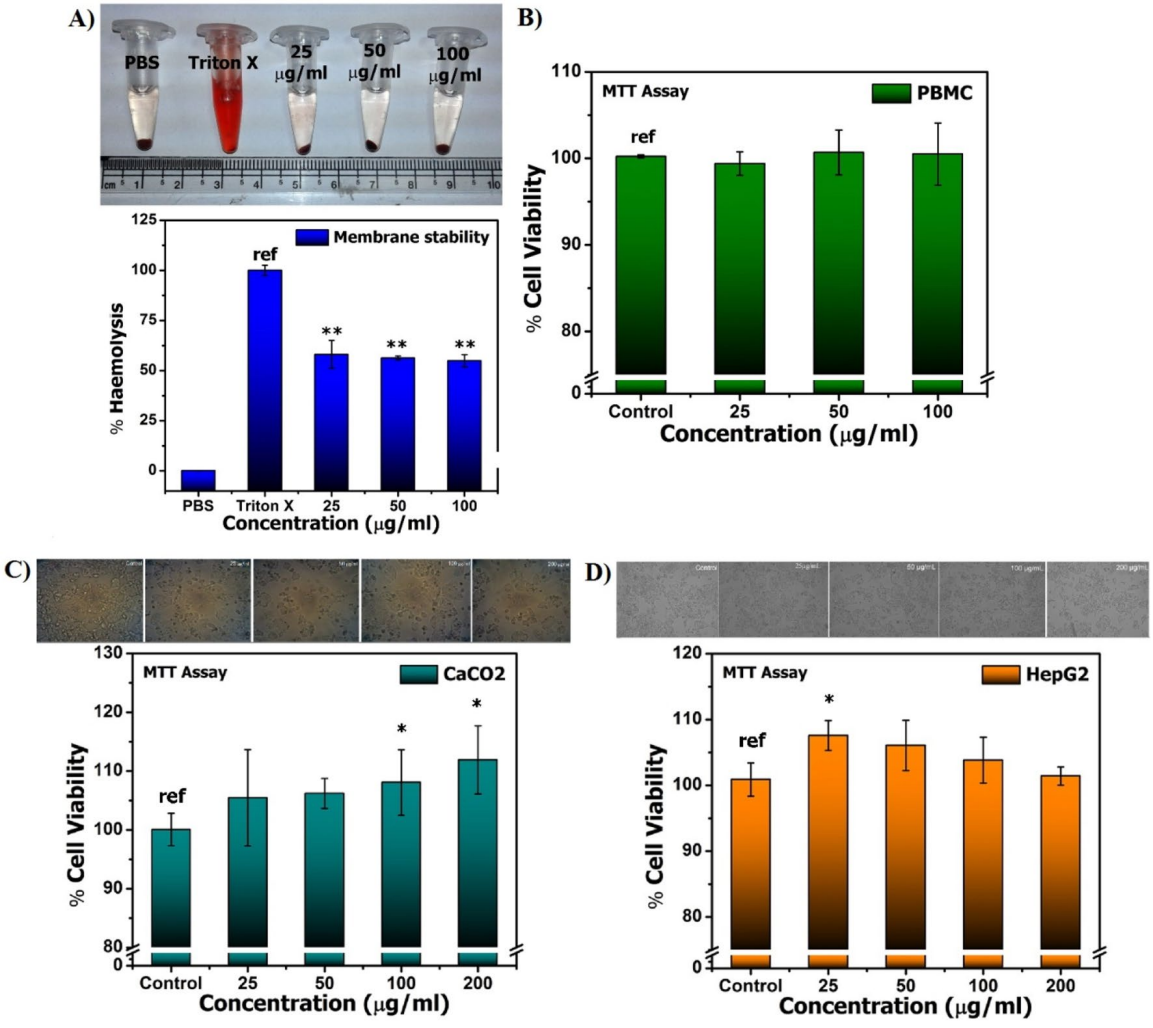
(**A**) Membrane stability assay of SAPE in erythrocytes and (**B**), (**C**) and (**D**) Results of Cell viability assay of SAPE at 48 h on cells of (**A**) PBMC, (**B**) Caco2 and (**C**) HepG2 cells with doses of (25, 50 and 100 µg/ml) or with 0.1% DMSO as the vehicle control measured by MTT based method. Graphs were presented in terms of mean ± SEM percentage of live, dead cells of the three independent sets. Data represented as mean ± S.E.M. Values were significant at ***P ≤ 0.001, **P ≤ 0.01 and *P ≤ 0.05 as compared to control.

The results for the membrane stability assay in terms of percentage of hemolysis are shown in Fig. 2A. It was found to be 0.0704% in case of the negative control PBS, and 100% in case of the positive control Triton X-100. Whereas, in case of SAPE it was found to be 58.08% for 25 µg/mL, and differed significantly at *P* ≤ 0.01 with the positive control; and 56.28% and 54.88% for 50 µg/mL and 100 µg/mL, respectively also differed significantly (*P* ≤ 0.001) with the positive control. Hence, it was observed that up to 200 ug/mL, the surface functionalization of SAPE conferred stability to the red blood cells and prevented hemolysis to a considerable extent. It was also observed that the membrane stability increased with increasing concentration of SAPE. Triton X-100 is generally used to lyse cells or to permeabilize the membranes of living cells. It was observed that in presence of SAPE, Triton X-100 exhibited significantly less hemolysis than the pristine samples.

The results for MTT assay of SAPE on PBMC, human intestinal cell lines derived from colon carcinoma (CaCo-2) and liver cancer cell lines (HepG-2) are shown in Fig. 2B–D respectively. Whereas no significant difference (*P* ≤ 0.05) in cell viability was observed in case of PBMCs, the results for the assay with CaCo-2 cells revealed that the percentage of viable cells increased with increasing concentration of SAPE. There was no any significant change in the number of viable cells after treating with 25, 50 and 100 µg/mL of SAPE, as compared to the control. However, at 200 µg/mL of SAPE a significant difference (*P* ≤ 0.05) was observed. Since CaCo-2 cells expresses several morphological and functional characteristics of the mature absorptive enterocytes with brush border layer as found in the small intestine^24,25^, and the results corroborates the safety of cellular uptake of SAPE. HepG-2 expresses most of the drug-metabolising enzymes^26^, and in the assay with HepG-2 it was observed that even though the percentage of viable cells decreased with increasing concentrations of SAPE, there was no significant change (*P* ≤ 0.05) in the number of viable cells after treating with varying concentrations of SAPE, as compared to the control.

### In vitro efficacy of SAPE in inhibition of COX-2

**Figure 3:**
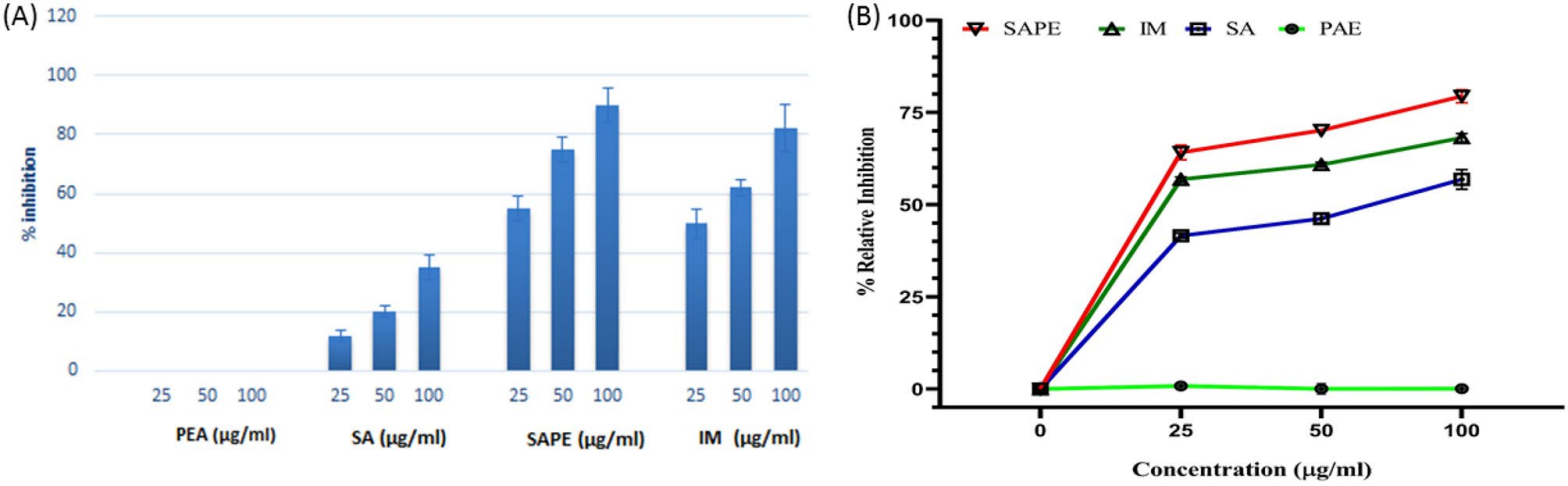
(**A**) The inhibition of COX-2 production in inflamed CaCo-2 cells, and (**B**) the direct relative inhibition of COX-2 by phenyl ethyl alcohol (PEA), salicylic acid (SA), salicylic acid phenyl ethyl ester (SAPE) and indomethacin (IM).

COX-2 is an inducible isoform of cyclooxygenase that is mainly produced in inflamed tissues and effectively absent in healthy tissue. It is induced in migratory and other cells by proinflammatory agents such as endotoxins, mitogens and cytokines under pathological conditions ^27^. Many anti-inflammatory, antipyretic, analgesic and antithrombotic effects are attributed to the inhibition of COX-2 activity by NSAIDs. In the LPS stimulated CaCo-2 cellular assay, the percentage inhibition of COX-2 production was calculated by considering the untreated stimulated cells as control and the results are shown in Fig. 3A. It was observed that phenylethyl alcohol alone did not possess any COX-2 inhibitory activity. The ester showed better COX-2 inhibition at all the studied concentrations (25, 50 and 100 µg/ml) than salicylic acid. Moreover, at these concentrations its COX-2 inhibitory activity was at par with indomethacin. In the direct inhibition assay also it was observed that the activity of SAPE was higher (Fig. 3B). At a concentration of 100 µg/ml, the percentage relative inhibition (79.42%) which was obtained by SAPE was higher than both salicyclic acid (68.13%) and indomethacin (65.69%). Moreover, the low IC_50_ value of SAPE (9.37 µg/ml) as compared to salicyclic acid (58.84 µg/ml) and indomethacin (12.69 µg/ml) is a clear indicative of the high dose efficacy of the ester under in vitro conditions.

From the results it becomes evident that the esterfication of salicylic acid considerably increases the anti-inflammatory activity of salicylic acid and these results corroborates with the previous findings^5,13,14^. The findings hold enormity because compounds that inhibit COX-2 activity finds importance both in the treatment of inflammatory responses and maintenance of human health and wellness. This is because aberrant expression of COX-2 has been widely implicated in the pathogenesis of many types of cancer types like colorectal cancer and its high levels have been associated with a decreased rate of cancer patient survival^28^.

### In vitro anticancer activity of SAPE upon MCF-7 cells

#### Cell viability assay

**Figure 4:**
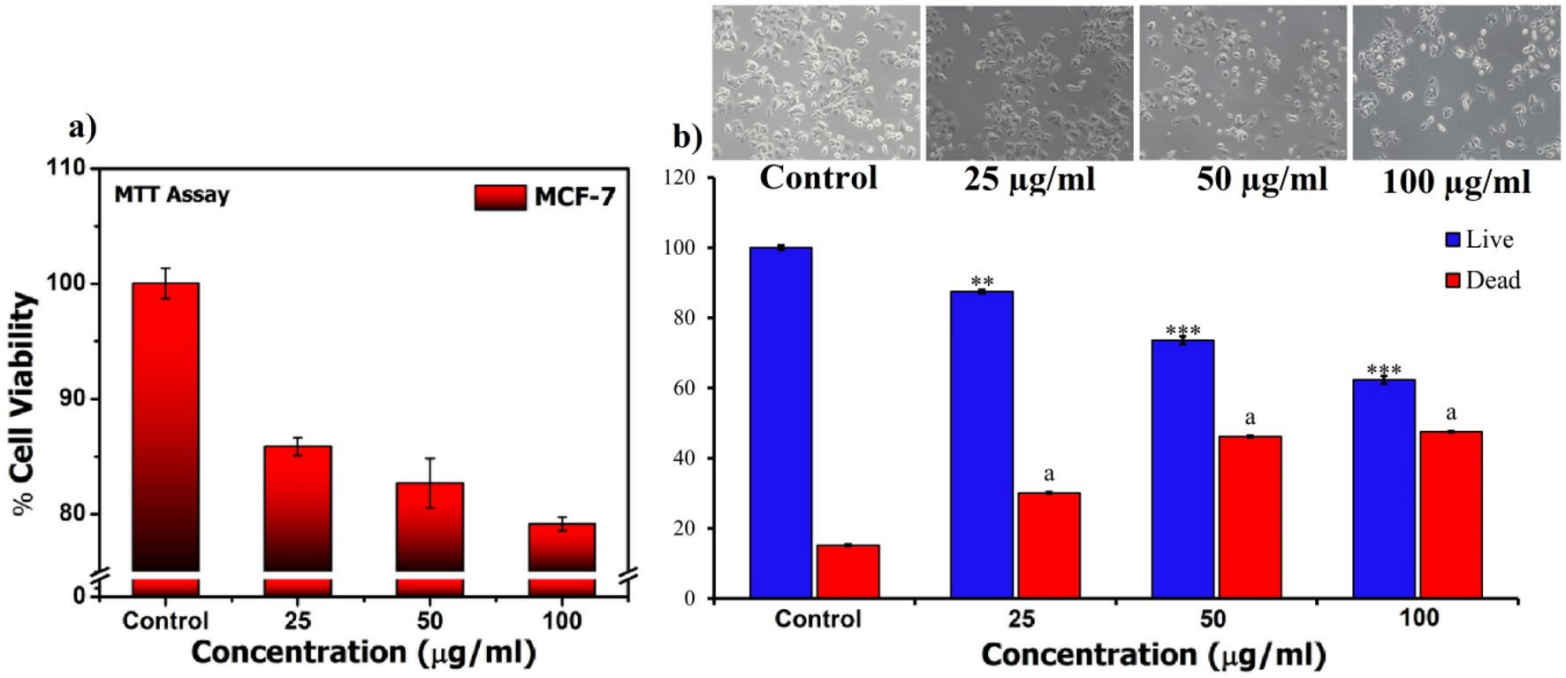
(**a**) Results of cell viability assay of SAPE on MCF-7 cells measured by MTT based method. (**b**) Cell viability assay of SAPE at 48 h on cells of MCF-7 with doses of (25, 50 and 100 µg/mL) or with 0.1% DMSO as the vehicle control using trypan blue exclusion method. Graphs are presented in terms of mean ± SEM percentage of live and dead cells of the three independent sets. Values were significant at *P ≤ 0.05, **P ≤ 0.01 and ***P ≤ 0.001 as compared to control.

To determine the cytotoxic effect of SAPE on MCF-7 cells, cell viability assay was performed using the MTT (Fig. 4a) and trypan blue dye exclusion (Fig. 4b) methods**.** Cells treated with various doses (25, 50 and 100 µg/mL) of SAPE were evaluated for its effect on live and dead cell numbers, which were calculated at the end of 48 h. It was observed that the decrease in total cell number is accompanied by an increase in significant cell death. The results clearly demonstrated that SAPE could significantly inhibit the exponentially growing MCF-7 cell viability with increasing concentration, while at the same time it did not have any pronounced effect on normal cells, as evident from the study with human PBMCs, Caco2 and HepG2 cells, thereby suggesting that the effect of SAPE was selective for cancer cells.

#### Cell cycle analysis

**Figure 5:**
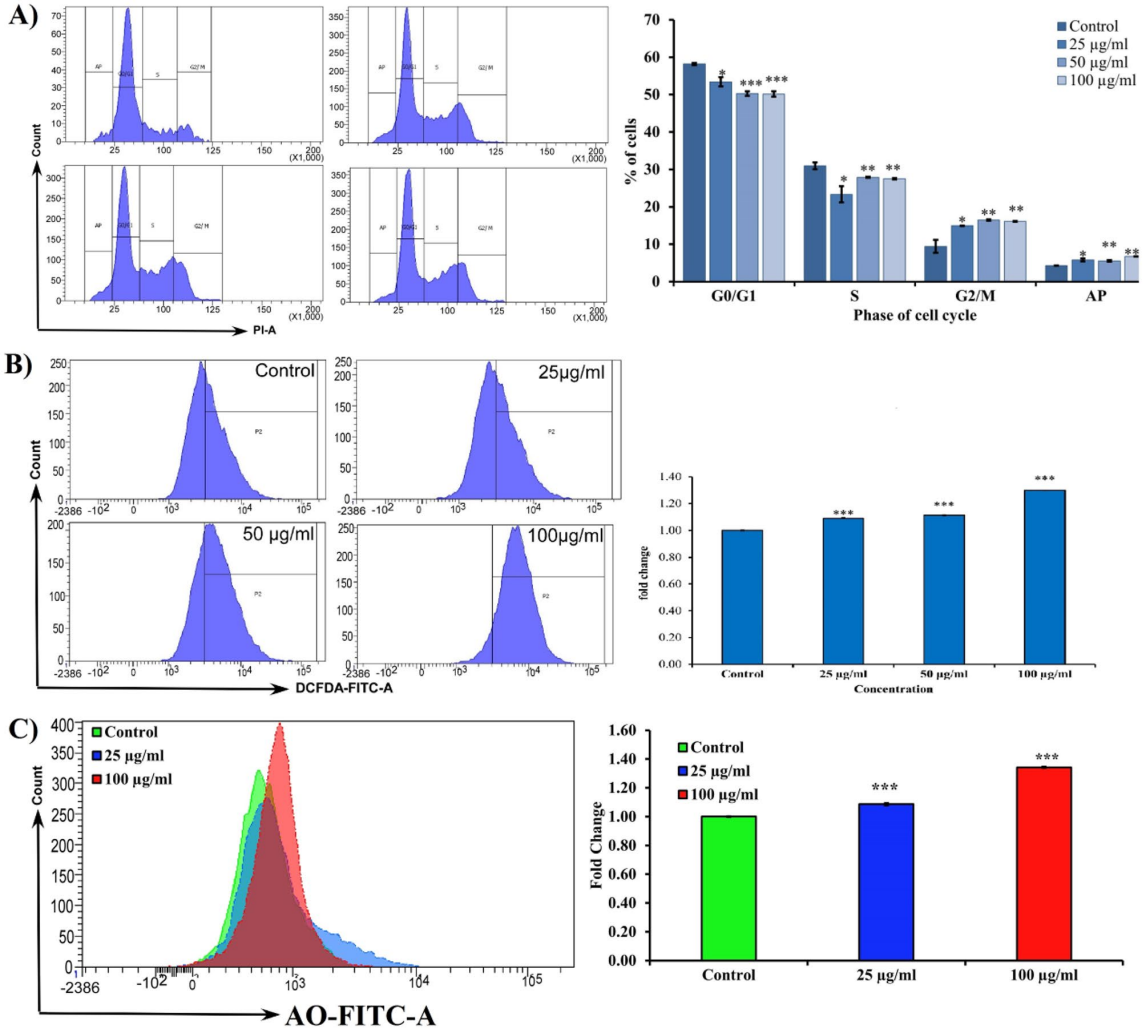
(**A**) Cell cycle analysis of SAPE on MCF-7 cells with doses of 25, 50, 100 µg/mL or with 0.1% DMSO as the vehicle control for 48 h of cell cycle phase distribution detected in a flow cytometer. Histogram display of DNA content viz. PI-fluorescence (x-axis) vs. counts (y-axis) and corresponding bar diagram is the representation of cell cycle phase distribution of G0/G1, S and G2/M phases. (**B**) Effect of SAPE on intracellular ROS levels in MCF-7 cells at 48 h were analyzed by flow cytometer with ROS specific fluorescence dye (DCFDA). Intracellular ROS levels were depicted through histogram and corresponding fold changes represented by bar graph were assessed by flow cytometry. (**C**) Effect of SAPE on MMP in MCF-7 cells. After that the fluorescent intensity of Rhodamine-123 was measured using flow cytometry at 488 nm (excitation) and 525–530 nm (emission) and data were depicted through histogram and corresponding fold changes represented by bar graph. Data represented as mean ± S.E.M. Values were significant at ***P ≤ 0.001, **P ≤ 0.01 and *P ≤ 0.05 as compared to control.

The MCF-7 cell line is an ER-positive and luminal subtype human breast carcinoma^29^. It was seen that treatment of MCF-7 cells with SAPE has a tendency towards alteration in the distribution of various cell cycle phases. As evident from Fig. 5A, treatment with SAPE led to a decrease in the percentage of cells in the G0/G1 and S phase, as compared to untreated cells. This indicates that SAPE results in induction of apoptosis, since the sub-G1 cells are actually the apoptotic cells with low DNA content. In the G2/M as well as the apoptopic phases, it was seen that treatment with SAPE causes a significant increase in the % of cells. Thus, SAPE induces a prominent G2/M arrest in MCF-7 cells in a dose dependent nature (*P* ≤ 0.001). However, when the dose of SAPE were increased up to 100 µg/mL, we found a significant decrease in the percentage of cells in the G1 phase, nonetheless, more cells also got arrested in the S phase as well as in the apoptotic phase (Fig. 5A) of the cell cycle. These results are suggestive of the inhibition of cell growth leading to increase in the number of cells entering in the G1 phase, which subsequently induces cell apoptosis.

#### ROS analysis

The level of ROS in the cells was analyzed in order to address the role played by ROS in SAPE induced apoptosis. It was observed that at the end of the 48 h of exposure to SAPE, ROS generation was induced in a significant manner, as evidenced by the increase in fluorescence intensity (Fig. 5B). In comparison with untreated cells, we found that SAPE caused increase in number of ROS positive cells, as well as mean fluorescence intensity (MFI) to 1.30 folds (*P* ≤ 0.001) for MCF-7, as shown in Fig. 5B. ROS is mainly produced in the mitochondria and an up gradation in the level of ROS could trigger mitochondrial-initiated events leading to apoptosis. Also, production of ROS could disrupt the homeostasis of the enzyme system of antioxidants responsible for ROS scavenging. Evidences suggest that ROS accumulation might be a result of increase in the ratio of Bax and Bcl-2, which decreases the MMP (as will be evident in “MMP analysis” section), and further induce the release of cytochrome C^58^. Results clearly demonstrate that SAPE could significantly elevate ROS level in MCF-7cells, and is an indicative of possible function of ROS in the apoptosis process.

#### MMP analysis

The loss in MMP was analyzed, since a loss in Δψm is directly associated with disruption of balance between the expressions of pro- and anti-apoptotic proteins within the Bcl-2 family. A reduction in Δψm leads to release of mitochondrial cytochrome C, thereby resulting in the formation of apoptosomes, which ultimately results in mitochondria mediated apoptosis^30^. The loss of the ΔΨm was reflected by a decrease in the intensity of rhodamine-123 fluorescent staining technique, which was used to detect mitochondrial membrane integrity or mitochondrial membrane depolarization. The results of the present investigation revealed that on exposure to SAPE, there was a significant decrease in mitochondrial membrane potential as compared to control. This was evidenced from the change in mean fluorescence intensity (MFI) which evinced a fold change increase to 1.34 folds (*P* ≤ 0.001) with highest doses of SAPE at 48 h (Fig. 5C). These results have distinctively shown that SAPE might induce apoptosis via mitochondrial pathways, and this could be a novel strategy for cancer therapy since mitochondria are one of the major pathways for apoptosis.

#### Apoptosis analysis

**Figure 6:**
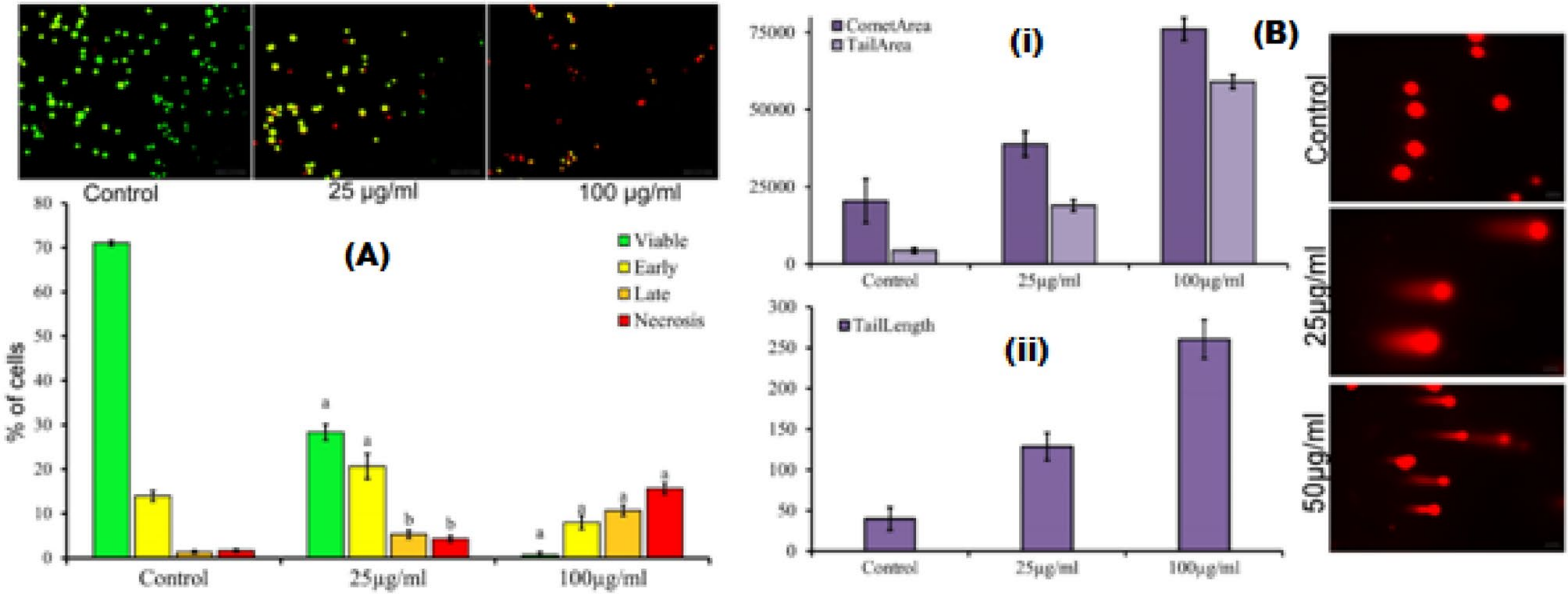
(**A**) Representative pictures of apoptosis assay of SAPE on MCF-7 with different doses (25, and100 µg/mL) or with 0.1% DMSO as the vehicle control for 48 h, determined by AO/EtBr dye staining; (**B**) Representative Comet images for DNA damage in of SAPE in MCF-7 cells detected by neutral comet assay, and quantification of the percentage of DNA damaged cells by measuring the (i) area of comet and (ii) length of comet tail with Open Comet software. Data represented as mean ± SEM. Values were significant at *P ≤ 0.05, **P ≤ 0.01 and ***P ≤ 0.001 as compared to control.

Apoptosis or type I programmed cell death is an inbuilt mechanism of the body to destroy cells that represent a threat to the physiological integrity of an organism. It is switched on by the imbalance between proapoptotic and antiapoptotic signals^31^. The main morphological change characterizing apoptosis is formation of apoptotic bodies, coupled with membrane blebbing, cellular shrinkage and chromatin condensation^30^. The results (Fig. 6A) showed that MCF-7 cells treated with SAPE at various doses exhibited gradient of color shift from green-orange-red when stained with dual AO/EtBr stain, signifying the effect of effective concentration to which cells were exposed with, whereas untreated cancer cells continued to fluoresce green in color. Thus, it could be interpreted that increasing concentration (25 and 100 g/mL) of SAPE significantly (*P* ≤ 0.001) induced damage to the cancer cells promoting cells to undergo apoptosis. This nuclear damage ranges from early apoptosis (found at low doses) to late apoptosis or necrosis as the dose increases (Fig. 6A). Thus, the induction of apoptosis in MCF-7 cells that make them more susceptible towards host phagocytosis without initiating inflammation could be attributed to the tumoricidal activity of SAPE.

Accessing the integrity of DNA is a useful marker for screening potential anticancer agent since the phenomena of apoptosis is expressed by cells after suffering DNA damage^31^. Moreover, induction of DNA damage might be an effective tool for cancer treatment. The results of the neutral comet assay presented in Fig. 6B showed the DNA damage in MCF-7 cells exposed to three different concentrations 25and 100 µg/mL of SAPE for 48 h found significantly (*P* ≤ 0.001) genotoxic to the respective cells, as noted by the concentration dependent increase in DNA fragmentation.The comet assay is an indicative of the level of heterogenic DNA damage within the individual cells, and the DNA damage in MCF-7 cells further demonstrates a potential tumour response factor for the compound^32^. Based on these observations, we found that SAPE could induce MCF-7 cell apoptosis. Anticancer agents that could modulate apoptosis are able to affect the steady state of cell populations, which is effective towards management and therapy of cancer. Hence SAPE could be speculated to have anticancer activity against MCF-7cells via induction of apoptosis.

#### Autophagy analysis

**Figure 7:**
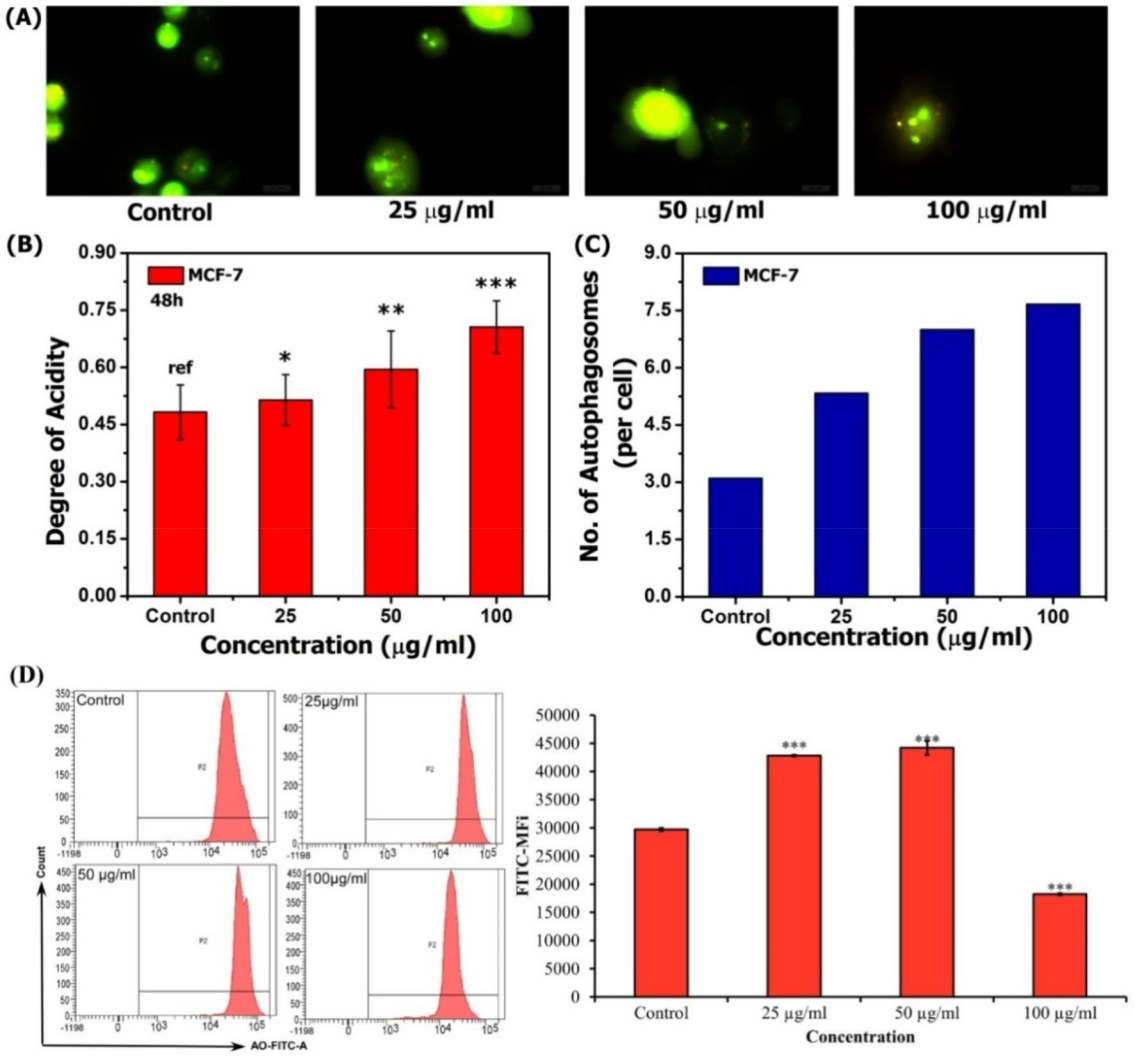
(**A**) Fluorescence microscopy for analysis of acidic vesicles and autophagosomes against SAPE treated MCF-7 cells (**B**) Analysis of the degree of acidity against SAPE (25, 50, 100 µg/mL) treated MCF7 cells for 48 h. (**C**) Analysis of autophagic vesicles formation against MCF7 cells treated with SAPE (25, 50, 100 µg/mL) for 48 h; (**D**) The mean fluorescent intensity of AO measured using flow cytometry at 488 nm (excitation) and 525–530 nm (emission). Data expressed as (Mean ± SEM, n = 3). Values were significant at *P ≤ 0.05, **P ≤ 0.01 and ***P ≤ 0.001 as compared to control.

Autophagy or type II programmed cell death is an evolutionarily conserved catabolic mechanism of cell death involving degradation of damaged proteins or organelles. It exerts a protective role which allows cancer cells to survive against cytotoxic agents. During autophagy, autophagosomes sequester cytoplasmic constituents and subsequently fuse with lysosomes to form autolysosomes, within which the engulfed organelles are degraded^29,30^. In order to ascertain whether autophagy inhibition enhanced SAPE-induced apoptosis in MCF-7 cells, the autophagy analysis was carried out. It was observed that SAPE exposure to MCF-7cells causes significant increase in both autophagic body and acid vesicles. We also visualized the effect of SAPE treatment on the formation of acidic vesicular organelles (AVOs) in MCF-7cells for 48 h using fluorescence microscopy upon staining with the lysosomotropic agent AO as shown in Fig. 7A. As evident, treatment of MCF7 cells with SAPE resulted in vacuole and condensed mitochondria formation, dispersed chromatin, apoptotic body formation, autophagic vesicles and membrane blebbing. Images obtained by fluorescence studies were processed with ImageJ to evaluate the degree of acidity as well as the number of autophagosomes. Whereas, untreated cells had normal nuclear and cytoplasmic morphology, it was observed that there was significant increase in the degree of acidity of cells at higher concentrations (Fig. 7B). Furthermore, the number of autophagosomes was also found to increase upon treatment, which might be due to increase in the degree of acidity, and this further reflects autophagic microenvironment within the cells.

For quantitative analysis, we quantitated AO fluorescence using flow cytometry. The formation of autophagic vacuoles was evidenced by increased red fluorescence intensity (Fig. 7C). It was observed that at the end of 48 h of exposure by SAPE treatment, autophagic vacuoles were induced significantly, as indicated by the increase in fluorescence intensity (Fig. 7D). In comparison with untreated cells, we found that SAPE treated cells with dosage range of 25-100 µg/mL caused increased MFi of autophagic vacuoles (1.48 folds at 50 µg/mL) in positive cells but at 100 µg/mL it decreased MFi to 0.613 folds as for 48 h. As evident from other studies^29,30^, autophagy plays role in the regulation of survival responses in malignant melanoma cells which have been targeted using different immunotherapeutic approaches, and thus these results evince a positive role of SAPE as an anticancer agent against MCF-7 cells.

### In vivo study for anti-inflammatory role of SAPE in animal model

#### Pre-test in paw oedema model

The results obtained for the test of acute inflammatory process by paw oedema model are presented in Table 3. A decrease in the % of paw oedema was observed in the SAPE treated group right from the 1st hour till the 5th hour. However, significant difference was observed only in the 3rd, 4th and 5th hour after carrageenan injection. This difference after the 3rd hour might be indicative of the action of SAPE that involves the inhibition of the synthesis or the release of prostaglandins. The absence of significant difference in the early hours might be due to the lack of influence of SAPE on the early mediators, viz., histamine, serotonin or bradykinin. Initially during the first hour after carrageenan injection, these early mediators are released, followed by the release of prostaglandins at around 3rd hour. The results are thus indicative that SAPE might act as inhibitors of cyclooxygenase enzymes involved in prostaglandins synthesis^33^.

**Table 3:** Effect of SAPE on the carrageenan induced paw oedema model.

Treatment	% of oedema at different time after carrageenan injection				
	1 h	2 h	3 h	4 h	5 h
Control	30.97 ± 2.25	26.33 ± 3.11	26.67 ± 2.26	22.61 ± 2.51	20.34 ± 2.22
SAPE	29.63 ± 2.99	25.32 ± 2.41	18.59 ± 3.09*	16.94 ± 3.44*	11.59 ± 3.14*

Results in mean ± SD (n = 4).

*Differ significantly from the control group (*P* ≤ 0.05).

#### Change in body weight of the different groups of rats and evaluation of disease activity index (DAI)

Initial weights of the rats are shown in Table ST3. Weight loss in animals may occur after treatment with non-steroidal anti-inflammatory drugs. This weight loss may result mostly from cachexia, the major cause of which may be due to cytokine excess^34^. However, as seen in Fig. SF12, a steady increase in the percentage of body weight with time was observed in all the three groups. The increase was however, higher in the control group as compared to the SAPE treated and indomethacin treated groups. The inflammatory bowel diseases (IBDs) like Crohn’s disease (CD) and ulcerative colitis (UC) are marked using the disease activity index (DAI) which help to evaluate disease activity at a given time^35^. The DAI scoring criteria and the DAI scores of the different treatment groups of rats are shown in Tables ST4 and Table ST5, respectively. It was seen that the colitis induced group without any treatment (RG-CI) marked the highest score for DAI with 2.42, whereas in the control group it was 0. Both the SAPE (1.04 DAI) and indomethacin (0.92 DAI) treated groups recorded lower scores than the RG-CI group. Hence there was a marked difference in the degree of induced disease after the rat models underwent pre-treatment with SAPE.

#### Intestinal morphological changes

**Figure 8:**
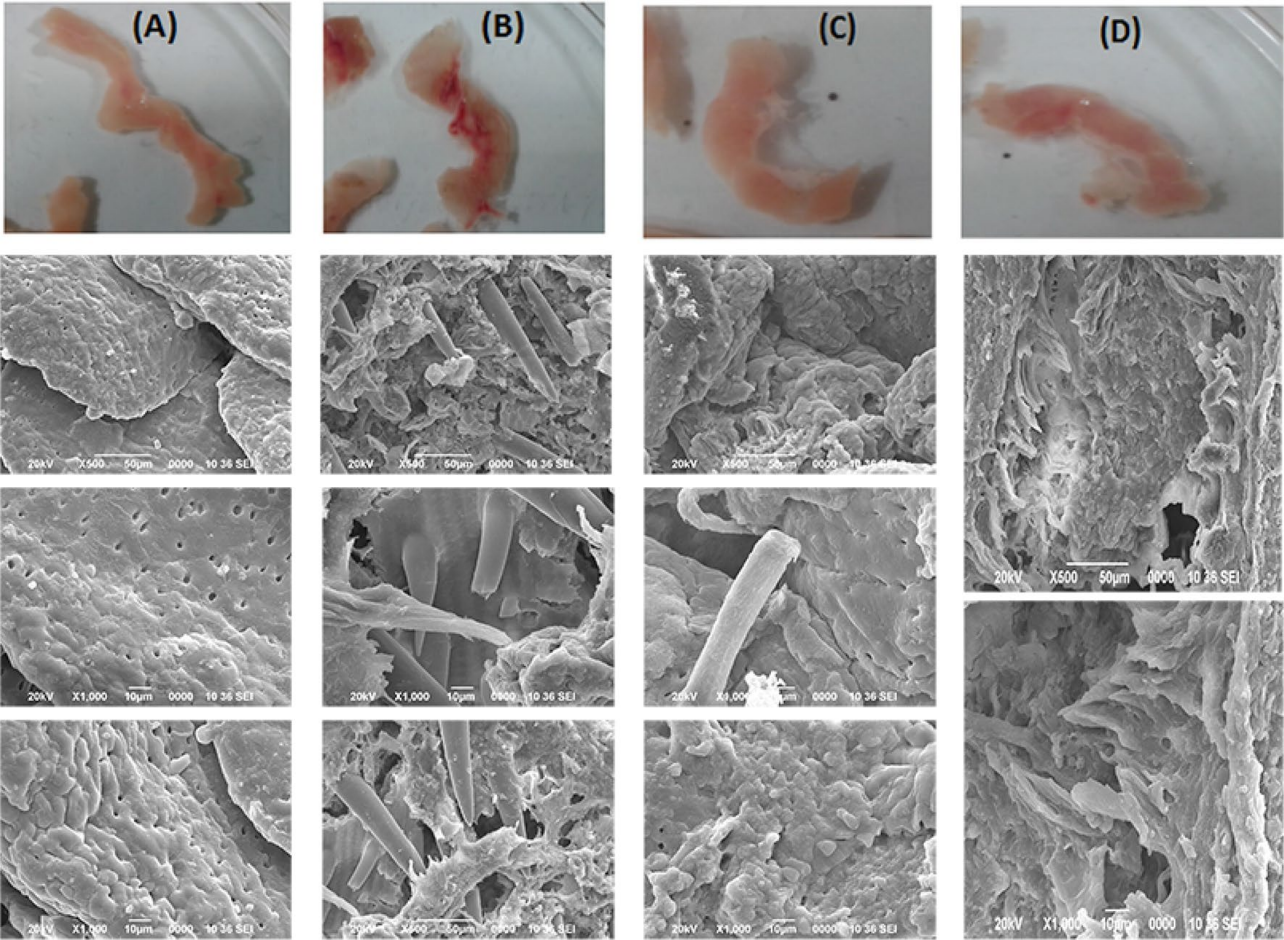
Excised intestines and SEM images of the intestinal section from (**A**) RG-CF (colitis free control group without any treatment) rats, (**B**) RG-CI (colitis induced group without any treatment) rats, (**C**) RG-SP (colitis induced group treated with SAPE) rats, RG-IM (colitis induced group treated with indomethacin) rats.

Excised intestines of the different groups of rats after treatment and euthanization, and their SEM images are shown in Fig. 8. Certain degree of tissue damage and rupture was observed in the colitis induced groups (RG-CI, RG-SP and RG-IM), which was not seen in the control group. As compared to the colitis induced group without any treatment, the SEM images for the SAPE and indomethacin treated groups displayed distinctive reduction in the level of tissue damage. This clearly indicates a marked reduction in the extent of colitis after treatment with SAPE.

#### Antioxidative and anti-inflammatory role of SAPE on animal model

##### Antioxidative effects

**Figure 9:**
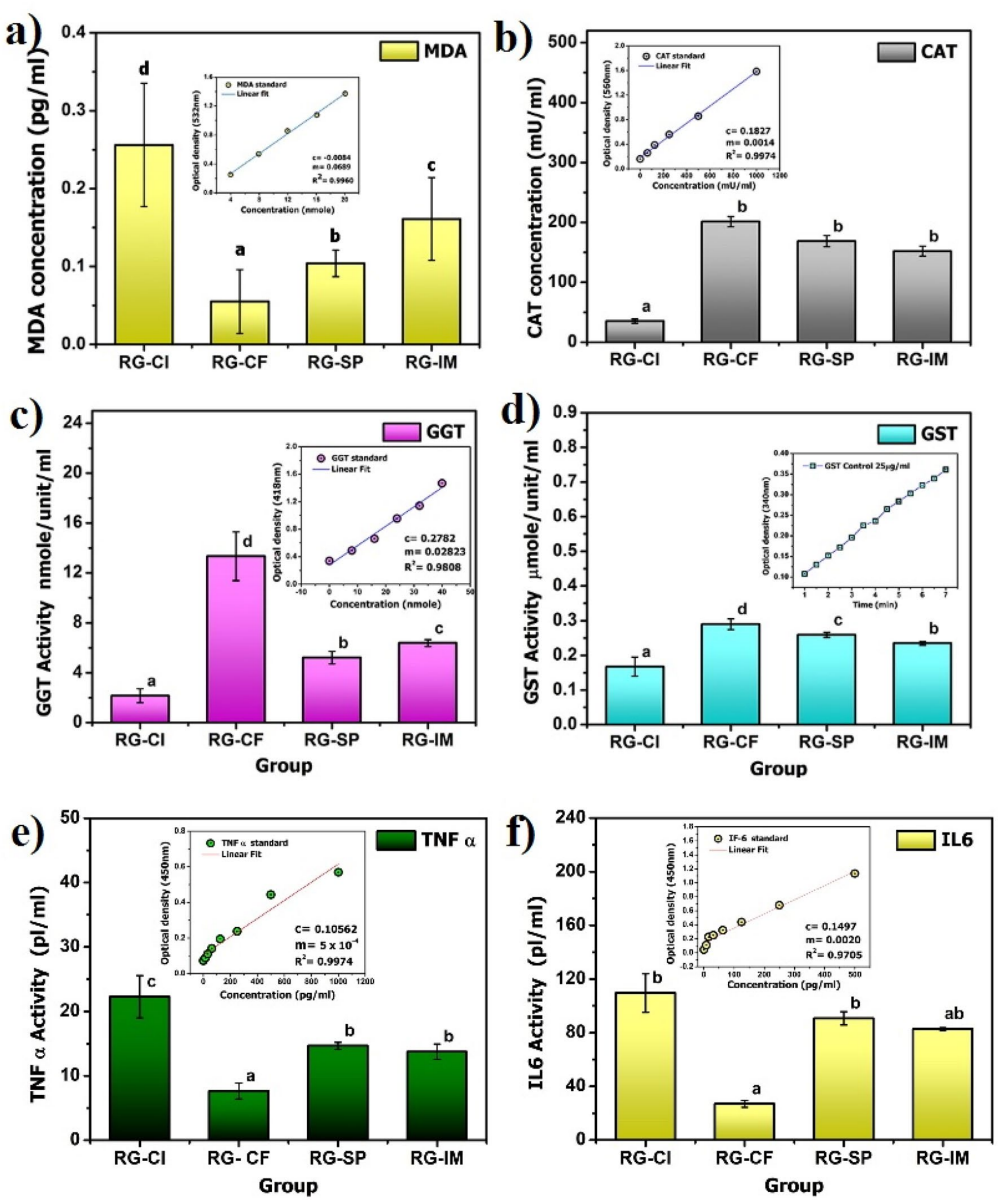
(**a**) Malondialdehyde (MDA) content; (**b**) catalase (CAT) content; (**c**) γ-glutamyl transferase (GGT) activity; (**d**) glutathione S-transferase (GST) activity; (**e**) tumor necrosis factor alpha (TNF-α) content and (**f**) interleukin 6 (IL-6) content in the intestinal tissue extract for different treatment groups of rats (RG-CI: colitis induced group without any treatment; RG-CF: colitis free control group without any treatment; RG-SP: colitis induced group treated with SAPE; Group RG-IM: colitis induced group treated with indomethacin).

Inflammation induced oxidative damage is aggravated by the decrease in antioxidant enzymes activities such as catalase (CAT), glutathione S-transferase (GST) and gamma-glutamyltransferase (GGT) which act as free radical scavengers in conditions associated with oxidative stress^36^. The polyunsaturated fatty acids, glycolipids, phospholipids, and cholesterol are well-known targets of peroxidative modification^37^ and the most common targets are components of biological membranes^38^. Lipid peroxidation produces a wide variety of secondary oxidation products, like the aldehydes, among which malondialdehyde (MDA) is the most mutagenic^37^. MDA is a low-molecular weight aldehyde that is generated as an end-product by decomposition of arachidonic acid and larger PUFAs, through enzymatic or non-enzymatic processes. Excessive MDA production has been associated with different pathological states and subjects affected by several diseases have increased levels of MDA. Hence, it is used as a convenient biomarker for lipid peroxidation by its facile reaction with thiobarbituric acid (TBA)^37,38^. Results of assay for MDA content in the intestinal tissue extract for different treatment groups of rats are illustrated in Fig. 9a. It was seen that there was significant difference (*P* ≤ 0.05) in the content of MDA among all the groups. The colitis induced group without any treatment showed the highest content (0.26 pg/µl), while the content was less in the SAPE treated group (0.10 pg/µl) as compared to the indomethacin treated group (0.16 pg/µl). Hence, SAPE could be considered as a potent inhibitor of lipid peroxidation in inflamed tissues. CAT is considered as a sensitive biomarker of oxidative stress in cells as it is a primary antioxidant defense component to protect cells from oxidative stress by eliminating toxic hydrogen peroxide from intracellular and extracellular environment^36,39^. Results of assay for catalase (CAT) concentration in the intestinal tissue extract for different treatment groups of rats are shown in Fig. 9b. It was seen that there was significant difference (*P* ≤ 0.05) in the content of CAT among all the groups. The colitis induced group without any treatment showed the lowest content (35.25 mU/mL), while the control group recorded the highest content (201.25 mU/mL). The content was again higher in the SAPE treated group (168.75 mU/mL) as compared to the indomethacin treated group (152.00 mU/mL), thus indicating a positive role of SAPE in the synthesis and activity of the antioxidative enzyme CAT.

GGT contributes to the extracellular catabolism of glutathione (GSH), as it catalyzes the transfer of the gamma-glutamyl moiety from conjugated GSH to acceptors such as amino acids and dipeptides. It also breaks down GSH into its constitutive amino acids and thus provides the rate-limiting amino acid cysteine de novo synthesis of GSH^40^. The levels of GGT in serum are determined by several factors such as inflammation, alcoholic liver disease, gallbladder and biliary tract diseases, plasma lipid/lipoproteins content, hypertension, hyperuricemia, diabetes and various medications^40,41^. Results of assay of GGT activity in the intestinal tissue extract for different treatment groups of rats are shown in Fig. 9c. It was seen that there was significant difference (*P* ≤ 0.05) in the content of GGT among all the groups. The colitis induced group without any treatment recorded the highest content (13.32 nmole/unit/mL). The content in both the SAPE treated group (5.21 nmole/unit/mL) and indomethacin treated group (6.39 nmole/unit/mL) was higher than control group (2.16 nmole/unit/mL). Alternatively, the GSTs are major phase II detoxification enzymes which catalyses the conjugation of electrophilic substrates to GSH, and also have other functions like peroxidase and isomerase activities, protecting cells against H_2_O_2_-induced cell death, and non-catalytically binding of endogenous and exogenous ligands. They result in the formation of GSH conjugates, which are eventually eliminated by several transport mechanisms such as ATP-dependent GS-X pump, among others^42,43^. Results of assay for GST activity in the intestinal tissue extract for different treatment groups of rats are shown in Fig. 9d. Significant difference (*P* ≤ 0.05) in the GST activity was observed among all the groups. The control group recorded the lowest activity (0.17 µmole/mL/min), while the colitis induced group without any treatment evinced the highest activity (0.29 µmole/mL/min). The activity was however, higher in the SAPE treated group (0.26 µmole/mL/min) than the indomethacin treated group (0.24 µmole/mL/min). The GGT and GST induction in cells occur as a protective adaptation against oxidants that are produced during normal metabolism or if oxidative stress increases due to any factor like the presence of ROS^44^, and the results obtained suggests a reduction in the level of oxidative stress with the application of SAPE in diseased condition.

##### Anti-inflammatory effects

The cytokines are stimulators of the production of acute phase proteins that are produced during inflammatory processes. These inflammation associated cytokines include interleukin-6 (IL-6), IL-1β, tumour necrosis factor-α (TNF-α), interferon-γ, transforming growth factor-β, IL-8, colony-stimulating factors and growth factors. Functional pleiotropy and redundancy are characteristic features of these cytokines^45,46^. TNF-α is a proinflammatory cytokine involved in the innate immune response^47^. Macrophages are the major producers of TNFα and on a cellular level they regulate a number of critical cell functions including cell survival, cell proliferation, differentiation, and apoptosis, which are mediated by the two transmembrane receptors, TNF-R1 and TNF-R2. In general, TNFα does not generally provoke cell killing, but promotes gene transcription and cell activation^9,48^. Under diseased condition, TNFα production and TNF receptor signaling regulates many facets of macrophage function. It plays a pivotal role in orchestrating the production of a proinflammatory cytokine cascade in many inflammatory diseases like Crohn’s disease, rheumatoid arthritis, atherosclerosis, sepsis, psoriasis, diabetes, and obesity. It is one of the most abundant early mediators in inflamed tissue as it is rapidly released after trauma, infection, or exposure to bacterial-derived LPS^48^. Results of assay for TNF-α content in the intestinal tissue extract for different treatment groups of rats are shown in Fig. 9e. The TNF-α content was lowest in the control group (7.65 pg/mL). The content of TNF-α in both the SAPE (14.66 pg/mL) and indomethacin (13.75 pg/mL) treated groups, was lower than the colitis induced group without any treatment (22.28 pg/mL).

IL-6 is a pleiotropic cytokine produced at the site of inflammation and plays a key role in the acute phase response, regulation of immune responses and hematopoiesis^49^. It is also involved in the regulation of metabolic, regenerative, and neural processes. Acute phase changes reflect the presence and intensity of inflammation and IL-6 is the chief stimulator of the production of most acute phase proteins^45^. IL-6 might be produced by a variety of cells following stimulation such as infection, trauma, or immunological challenge. It has also been found to have a protective role in the LPS-galactosamine septic shock model in mice^49^. Results of assay for IL-6 content in the intestinal tissue extract for different treatment groups of rats are shown in Fig. 9f. Its content was lowest in the control group (26.91 pg/mL). There was no significant difference in content between the SAPE (90.76 pg/mL) and indomethacin (82.83 pg/mL) treated groups, and both revealed much lower content than the colitis induced group without any treatment (109.56 pg/mL). The results of both the IL6 and TNF- α assays clearly indicated a decrease in the level of inflammation after treatment with SAPE.

## Conclusion

Zn(OTf)2-catalyzed selective esterification was successfully employed for the production of salicylic acid phenylethyl ester (SAPE). This ester was found to be non-cytotoxic, with promising anti-inflammatory and antioxidative effects, and the results were at par with established NSAID agents. The ester exhibited anticancer activity against MCF7 breast cancer cells via sub-G1 cell cycle arrest, MMP reduction, ROS accumulation and induction of apoptosis and autophagy. The results for the first time provide an insight into the use of this particular ester as a potential anti-inflammatory and cancer therapeutic agent. Further research elucidating the cellular uptake and metabolism of SAPE would help to corroborate this ester as an NSAID.

## Materials and methods

### Materials

Human blood sample for obtaining primary peripheral blood mononuclear cells (PBMCs) was collected voluntarily in the Health Centre of Tezpur University, Assam, India. The embryonic human liver cancer cell line (HepG2), epithelial colorectal adenocarcinoma cell line (CaCo-2) and epithelial breast cancer cell line (MCF-7) were obtained from National Centre for Cell Science (NCCS), Pune, India. The media for animal cell culture were purchased from HiMedia, India and the chemicals and solvents were obtained from Sigma Aldrich, USA and Merck, Germany.

### Synthesis of salicylic acid phenylethyl ester (SAPE)

#### Zn(OTf)_2_-catalyzed selective esterification of salicylic acid and phenylethyl alcohol

Salicylic acid (1.0 equiv) under N_2_ atmosphere was added to a stirring solution of I_2_ (2.0 equiv) and triphenylphosphine (2.0 equiv) in dry acetonitrile (10 mL) and the reaction mixture was vortexed for 10 min, and then Zn(OTf)_2_ (5 mol %) was added. Stirring was continued for 30 min at 60 °C, and then added phenylethyl alcohol (1.1 equiv) in dry acetonitrile. After completion of the reaction (monitored by TLC), the mixture was cooled to 30ºC and the solvent was removed under reduced pressure. The residue was dissolved in ethyl acetate and washed with saturated NaHCO_3_ followed by brine solution. The organic layer was dried over anhydrous Na_2_SO_4_ and concentrated under vacuum. Column chromatography with silica gel and a gradient solvent system of ethyl acetate to hexane yielded the target compound^50^ and validated as mentioned in “NMR and FTIR study for structural validation of SAPE” section.

#### NMR and FTIR study for structural validation of SAPE

The ^13^C and ^1^H NMR studies were conducted in an NMR spectrophotometer (ECS-400, JEOL, Japan) and the software used for data acquisition was Delta, Ver. 4.3.6. The test compounds were dissolved in NMR grade chloroform and the field strength was set at 9.389766 [T] (400 MHz). The FTIR readings were taken in an FTIR spectrophotometer (Spectrum 100, Perkin Elmer, USA). The data were collected in absorbance (A) mode and the wavelength range selected was 4000 to 400 cm^−1^. The resolution and the number of scans per sample were 4 cm^−1^ and 4, respectively.

### In silico studies for SAPE’s activity against cycloxygenase-2 (COX-2)

#### Chemical structure generation and absorption, distribution, metabolism, excretion and toxicity (ADME-Tox) studies

For molecular docking simulation, the structure of SAPE, indomethacin and ibrupofen were generated using ChemOffice 2010 (Cambridge Soft, Massachusetts, USA). The compounds were then converted to 3D format for docking simulations and their geometries were optimized using MM2 force field methods^51^ and saved as sybyl mol2. The in silico ADME-Tox tests related to SAPE was carried out using the software ACD/Labs I-Lab 2.0 (Advanced Chemistry Development Inc, Toronto, Canada).

#### Molecular docking against COX-2

Molecular docking of SAPE, and two other established anti-inflammatory agents viz., indomethacin and ibrupofen was carried out against COX-2 (PDB ID: 4PH9) using Molegro Virtual Docker (MVD) 6.01 (CLC bio, Aarhus, Denmark). A grid based cavity prediction algorithm was used by employing a discrete grid of 0.8 Å resolution to cover the protein, followed by placing a sphere of 1.4 Å radius. The sphere was checked for overlaps with other spheres and the cavities found were ranked according to their volume^21^. For docking simulations, the bond and side chain were set with a tolerance of 1.7 and strength of 0.7 and the root mean squared deviation (RMSD) threshold for the multiple cluster poses were set at 2.00 Å. The docking algorithm was set at 1,500 maximum iterations, 50 simplex evolution size and minimum 100 runs for each compound. The molecular poses were ranked on the basis of MolDock and Rerank score, visualized and the top docking hits were selected for the ligand–protein interaction analysis.

#### Molecular dynamics (MD) simulation study of the docked complex

MD simulation studies were carried for the top docking hits against the protein–ligand docked complex. The ligand topology and atom charges were generated using the PRODRG server^52^, whereby the charge value was set to full while the chirality was set to default value with no further energy minimization. The systems were processed using Gromos 43a1 force field in which the protein–ligand system was immersed in water and energies were minimized, followed by NVT (canonical) and NPT (isothermal–isobaric) equilibrations. The equilibrated structures were subjected to 20 ns MD simulation, and the trajectory was analysed and plotted for RMSD backbone.

#### Density functional theory (DFT) study of the docked complex

The conformation of the best docking pose SAPE was exported and DFT calculations were carried out using Gaussian 09. Theoretical calculations were carried out at Ground State using DFT/B3LYP/6–31 basis set. The guess method was set as extended Huckel with mix HOMO and LUMO orbital. The molecular orbital energies were considered for calculating the band energy gap ΔE_*LUMO-HOMO*_*.*

### Test for cytotoxicity activity of SAPE

#### Culture of PBMCs, HepG-2, CaCo-2 and MCF-7cells

The necessary ethical committee permission for use of human blood was obtained from the Tezpur University Ethical Committee (TUEC), Tezpur University, Tezpur (Protocol no. DoRD/TUEC/10-14/4361 dated 28.03.2014). Preinformed consent was obtained from all the volunteers to donate blood for obtaining the peripheral blood mononuclear cells (PBMCs) and red blood cells (RBCs) needed for this experiment. All the cells were grown in a stable environment with 5% CO_2_ at 37 °C. PBMCs were isolated by density gradient centrifugation and seeded in RPMI-1640 media (3 × 10^3^/200µL) supplemented with 10% FBS in 96 well plates. CaCo-2 cells were cultured in MEM medium containing 20% fetal bovine serum, HepG-2 cells were cultured in DMEM medium and MCF-7 cells were cultured in MEM medium containing 10% FBS, 100 U/mL penicillin, and 100 mg/L streptomycin.

#### Membrane stability assay

The RBCs were isolated from the blood, washed with PBS (pH 7.4), centrifuged at 2000 rpm for 10 min and then 2% of the erythrocyte suspension (ES) was re-suspended in saline solution. The reaction mixture contained 100 μL of ES, 0.1% Triton X-100 and 25, 50 or 100 µg/mL of SAPE in 96-well microplates and incubated for 60 min at 37 °C under constant agitation and then centrifuged at 2000 rpm for 10 min. The release of haemoglobin was determined by photometric analysis of the supernatant at 576 nm. Used 0.1% Triton X-100 (as positive control) and PBS (as negative control) to achieve 100% and 0% hemolysis, respectively^53^.

#### 3-(4,5-dimethyl-2-thiazolyl)-2,5- diphenyl-2-H-tetrazoliumbromide (MTT) assay

The PBMC, HepG-2, CaCo-2 and MCF-7 cells (2 × 10^3^) were seeded in 96-well plates and treated with increasing concentrations (25, 50, 100 and 200 µg/mL) of SAPE for 48 h at 37 °C, 5% CO_2_. Thereafter, 20 µL (5 mg/mL in PBS) of MTT solution was added to each well and incubated for 4 h at 37ºC. After removing the media, formazons were dissolved in DMSO and optical density was measured at 570 nm. The cell viability inhibitory ratio was calculated in relation to control cells cultured in drug-free media^54^.

#### Trypan blue exclusion staining assay

The effect of SAPE on the viability of MCF-7 cells was also assessed by the trypan blue dye exclusion assay. The cells (2 × 10^3^) were cultured with different concentrations (25, 50 and 100 µg/mL) of SAPE in 0.1% DMSO and incubated for 48 h. A total of 0.2 ml of trypsinized cell solution was then mixed with 0.5 mL 0.4% trypan blue in PBS, and after 5 min the stained (dead) and unstained (viable) cells were counted using a hemocytometer^31^.

### In vitro inhibitory activity of SAPE against COX-2 enzyme

The compounds viz. SAPE, salicylic acid and phenylethyl alcohol (25, 50 and 100 µg/mL) were tested for their efficacy in inhibiting COX-2 production and compared with indomethacin. In the stimulated cellular assay, the CaCo-2 cells were pre-incubated in MEM media as mentioned previously ("Culture of PBMCs, HepG-2, CaCo-2 and MCF-7cells" section) in the presence and absence of the test compounds for 1 h. Lipopolysaccharides (LPS) from *Escherichia coli* 0111: B4 (10 µg/mL) was then added to the cell suspension and further incubated for 48 h at 37 °C and 5% CO_2_. After removing the media, the cells were washed with PBS, added into ice-cold cell extraction buffer (0.1 g/ml) and lysed using a bead beater (607EUR, BioSpec Products, USA). The supernatant of the tissue lysate was collected by centrifugation (12,000 × g for 10 min at 4 °C) and the release of Cox-2 was measured using a human COX-2 ELISA kit (RAB1034, Sigma-Aldrich, USA) by following the manufacturer’s instructions and readings were taken in a microplate reader (GloMax Explorer, Promega, USA).

For the direct sequestration assay of COX-2, a fluorimetric COX-2 Inhibitor Screening Kit (MAK399, Sigma Aldrich, USA) was used. The assay was based on the fluorometric detection of prostaglandin G2, the intermediate product generated by the COX enzyme. All the assay parameters were carried out according to the manufacturer’s instructions and the fluorescence was measured immediately in kinetic mode using a microplate reader at λ_Ex_ = 535 nm/λ_Em_ = 587 nm at 25 °C for 5–10 min. The IC_50_ values for each of the compounds were calculated using the software CompuSyn (ComboSyn, Inc.).

### Flow cytometry analysis (FCA) for anticancer activity of SAPE in MCF-7 cells

#### Cell cycle analysis

MCF-7 cells at 1 × 10^5^ cells/well were treated with various concentrations (25, 50 and 100 μg/mL) of SAPE. After 48 h of incubation, both adherent and floating cells were collected by trypsinization, washed twice with ice-cold PBS, fixed in ice-cold 70% methanol for overnight at -20ºC and subsequently incubated with propidium iodide (20 µg/mL) and RNase A (200 µg/mL) for another 30 min at 37ºC. Cell cycle distribution was then analysed by flow cytometer (BD FACS LSR III, BD Biosciences, USA). Finally, percentage of cells in different phases of cell cycle was determined by BD Diva software.

#### Reactive oxygen species (ROS) evaluation

For the quantitative detection of intercellular ROS generation, MCF-7 cells were seeded at a density of 1 × 10^5^ in 6 well culture plates for 24 h, followed by treatment with various concentrations (25, 50, and 100 μg/mL) of SAPE for 48 h. Subsequently the cells were trypsinised and washed with PBS and stained with 25 mM dichloroflourecien diacetate for 30 min at 37ºC in the dark and relative ROS levels of the cells were quantified by FCA ^30^.

#### *Mitochondrial membrane potential* (Δψm) *analysis*

The treatment of MCF-7 cells, as mentioned in previous "Cell cycle analysis" section, was followed by incubation with 400 μl of 50 μM Rhodamine 123® at 37ºC for 30 min with vertical shaking at intervals of 5 min. The cells were then washed thrice with PBS and the fluorescence intensity of Rhodamine 123® was measured by FCA with excitation and emission wavelengths fixed at 488 nm and 525–530 nm, respectively. The mean fluorescence intensity (MFI) hence represents the cellular levels of intracellular mitochondrial membrane potential (MMP) ^30^.

#### Apoptosis analysis

As mentioned in ***2.5.1***, MCF-7 cells (1 × 10^5^ cells/mL) were treated with various concentrations (0, 50, and 100 μg/mL) of SAPE. Thereafter cells were centrifuged at 3000 rpm for 5 min at 15ºC and the pellet was washed twice with PBS. The pellet was resuspended in 50 µL of PBS and out of the suspension, 25 µL was mixed with 2 µL of acridine orange (AO) and ethidium bromide (EB) mix (100 µg/mL-1:1) for 10 min and kept in an incubator at 37 °C. The cells were then mounted on a glass slide with cover slip and viewed under a fluorescent microscope (Leica DM300, Germany)^30^.

#### Autophagy analysis

For the qualitative analysis of intracellular autophagy by fluorescence microscopy^29^, imaging was achieved by seeding the cells on a cover-slip-loaded 6-well plate at 1 × 10^5^ cells/well. Thereafter, the cells were treated with SAPE and processed as indicated in ***4.6.1.*** Finally, after washing with PBS, the cells were stained with AO, and mounted onto a microscope and images were captured using appropriate filter settings in a compound microscope (Leica DM3000, USA).

Subsequently, as in 2.5.1, MCF-7 cells (1 × 10^5^ cells/mL) were treated with various concentrations (0, 50, and 100 μg/mL) of SAPE. Herein, the media was removed and the cells were stained with 1 μg/mL of AO at 37ºC for 15 min, followed by washing with PBS and immediately analysed by FCA.

### Comet assay for accessing DNA damage

This was done according to Olive and Banáth^32^. Briefly, 0.4 mL of MCF-7 cells (2 × 10^4^ cells/mL) was mixed with 1.2 mL of 1% agarose at 40 °C, and 1.2 mL of this cell suspension was settled onto the agarose-covered surface of a pre-coated (1% agarose) slide. The slides were then gently submerged in lysis solution (2% sarkosyl, 0.5 M Na_2_EDTA and 0.5 mg/ml proteinase K, pH 8.0) at 4ºC and incubated at 37ºC for 20 h in dark. It was then submerged in electrophoresis buffer solution (pH 8.5) for 30 min and subjected to electrophoresis in the same buffer for 25 min. Staining was done with 2.5 µ/mL propidium iodide in distilled water and the cells were analysed (50 comet images per slide) using Open Comet software by examining individual comet images for the area of comet and length of comet tail.

### Test for anti-oxidative and anti-inflammatory activity of SAPE in Wistar rats

The animal studies were conducted with albino rats (*Rattus norvegicus* of Wistar variety) at Defense Research Laboratory, Tezpur (CPCSEA Reg. Number: 1127/bc/07/CPCSEA), Assam. The necessary ethical committee permission for conducting the animal studies was obtained from the Institutional Animal Ethical Committee (IAEC), Defense Research Laboratory, Tezpur (Protocol no. 01/May/2016 dated 03.06.2016). All the experiments were performed in accordance with relevant guidelines and regulations. Moreover, the reporting of the studies involving rats followed the recommendations as stated in the ARRIVE guidelines.

#### Experimental design

The rats (2 months old) were housed in (20 ± 1)°C, (60 ± 5)% relative humidity and light/dark (12 h:12 h) cycle in specific pathogen-free barrier conditions. Rodent diet and water were given ad libitum. A pre-treatment model of drug administration was used and they were divided into five groups in a randomized manner with equal number of males and females. Group RG-CI: colitis induced group without any treatment (n = 8); Group RG-CF: colitis free control group without any treatment (n = 8); Group RG-SP: colitis induced group treated with SAPE (n = 8); Group RG-IM: colitis induced group treated with indomethacin (n = 8) and RG-PO: paw oedema induced model group (n = 8).

#### Pre-test in paw oedema model

Paw oedema was induced in a different group of rats (n = 8) by injecting 0.15 mL of 0.1% carrageenan into the sub-plantar region just below the lateral malleolus of both left paws. SAPE and indomethacin (10 mg/kg body weight) were then administered by a trans-dermal route. The paw volume was measured at 0, 1, 3 and 5 h using a digital plethysmometer (Orchid Scientific, India) and the % oedema was expressed according to Upadhyay et al.^55^.

#### Administration of drug and induction of colitis

Starting from day 1 till day 16, SAPE and indomethacin were fed to all members of the respective groups (Group RG-SP and RG-IM) at dosages of 250 mg and 1.5 mg, respectively per kg body weight. After 4 h elapse of the last dose, rats of group RG-CI, RG-SP and RG-IM were challenged with intraperitoneal injection of LPS from *Escherichia coli* 0111: B4 at a dose of 5 mg/kg body weight. All the rats were euthanized after 24 h of LPS challenge by CO_2_ asphyxiation. Post mortem, colons and small intestines were dissected and flushed with cold 1 × PBS containing penicillin/streptomycin (100 I.U/mL penicillin and 100 μg/mL streptomycin) with a protease inhibitor cocktail, and then opened lengthwise and washed extensively with PBS. Intestinal segments (0.5–1.0 cm), 2 cm away from the anus were collected and fixed in 10% neutral buffered formalin. The tissues were stored in pyrogen/endotoxin-free tubes and kept frozen at –80 °C prior to analysis.

#### Change in body weight and evaluation of disease activity index (DAI)

The body weights of all the rats were recorded every day before the treatment and the rate of body weight gain was calculated. The DAI scores were calculated by adding combined scores for weight loss, stool consistency, and faecal bleeding, and dividing this sum by 3^56^.

#### Scanning electron microscopy (SEM) of intestinal sections

The tissues were fixed with 3% glutaraldehyde, washed with 0.1 M sodium cacodylate buffer, and again fixed with 0.1% osmium tetroxide in 0.1 M sodium cacodylate buffer. Dehydration was done in series of 30–100% acetone, followed by drying in tetramethylsilane at 4 °C. The microstructures were investigated using a scanning electron microscope (JEOL JSM-6390LV, SEM, Oxford) at magnifications of 500 × and 1000 × and accelerating voltage of 20 kV.

#### Antioxidant activity assays of the intestinal tissues

The level of lipid peroxidation was expressed as the amount of malondialdehyde (MDA) formed, and this was analysed by using a lipid peroxidation assay kit (MAK085, Sigma-Aldrich, USA) which uses a standard curve of MDA ranging from 0 to 20 nmole. The content of catalase (CAT) was determined using a catalase assay kit (A22180, Invitrogen, USA), and the catalase concentration was calculated from a standard curve of catalase (0 to 4.0 U/mL) in which 1 unit of enzyme was defined as the amount that will decompose 1.0 μmole of H_2_O_2_ per minute at pH 7.0 and 25 °C temperature. The assay for γ-glutamyltransferase (GGT) activity was performed using a GGT assay kit (MAK089, Sigma-Aldrich, USA) and a pNA standard curve (0 to 40 nmole/well), where one unit of GGT was defined as the amount of enzyme that will generate 1.0 µmole of *p*NA per minute at 37 °C. The glutathione S-transferase (GST) activity assay was performed using the GST assay kit (CS0410, Sigma-Aldrich, USA), and the GST specific activity was calculated by using a molar extinction coefficient of 1-chloro-2,4-dinitrobenzene (CDNB)-GST conjugate.

#### Analysis of pro-inflammatory cytokines in the intestinal tissues

The tissues were homogenized by sonication with RIPA buffer and 1 mM PMSF. The lysate was centrifuged at 13,000 × g and the supernatant was further diluted 1:10 to 1:100 times with standard diluent buffer prior to analysis. The assay for interleukin 6 (IL-6) was performed using the mouse IL-6 ELISA kit (KMC0061, Invitrogen, USA) and an Ms IL-6 standard curve (0–250 pg/mL). Tumor necrosis factor alpha (TNF-α) was assayed by using the mouse TNF-α ELISA kit (KMC3011, Invitrogen, USA) and a Ms TNF-α standard curve (7.8–250 pg/mL).

### Statistical analysis

All the results were presented as mean ± SEM. Statistical analysis was performed using ANOVA following Mann–Whitney *U*-test. A value of *P* < 0.001, *P* < 0.01 and *P* < 0.05 were considered to indicate a significant difference between groups.

## Supplementary Information

Supplementary Information.
