# Supplementary material for: Synthesis of salicylic acid phenylethyl ester (SAPE) and its implication in immunomodulatory and anticancer roles
Source: Sci Rep. 2022 May 24;12:8735. doi: 10.1038/s41598-022-12524-7 (PMC9130252; doi:10.1038/s41598-022-12524-7)
Supplement: Supplementary file 1 — Supplementary Information. [file 41598_2022_12524_MOESM1_ESM.docx]

**Supplementary material**

**Figures:**





Fig SF1: The reaction for the production of SAPE


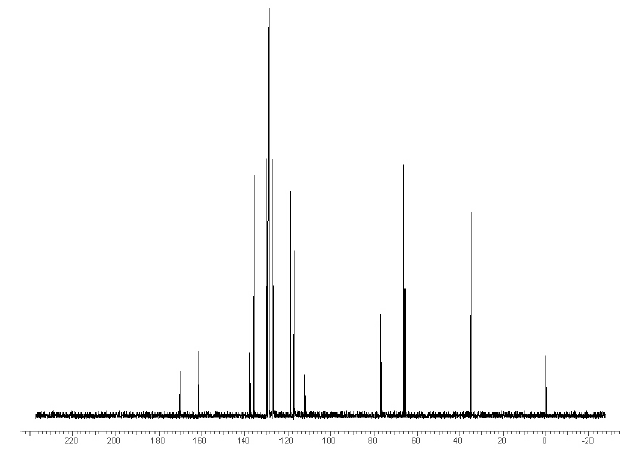


Fig SF2: C13 NMR of the synthesized SAPE molecule


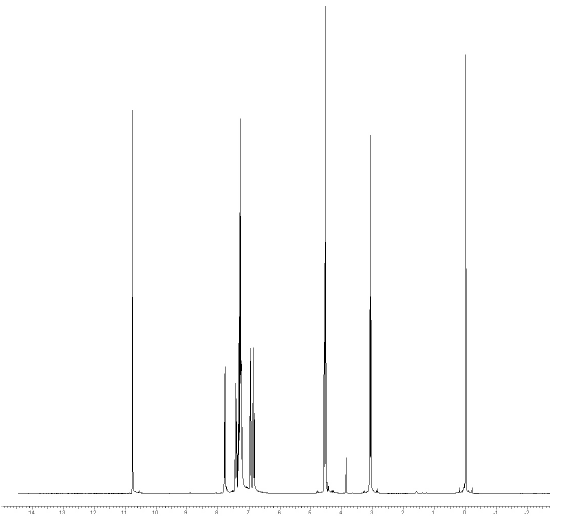


Fig SF3: H1 NMR of the synthesized SAPE molecule


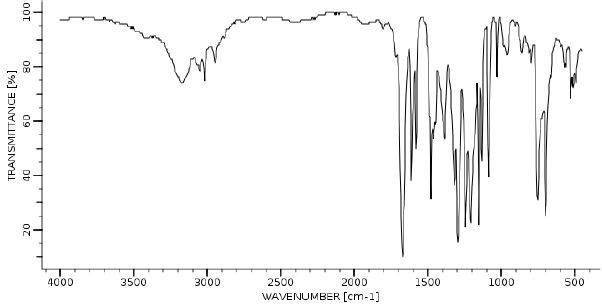


Fig SF4: FTIR plot of the synthesized SAPE molecule


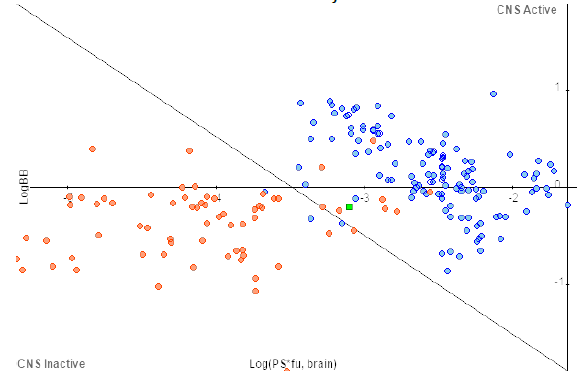


Fig SF5: CNS activity of SAPE


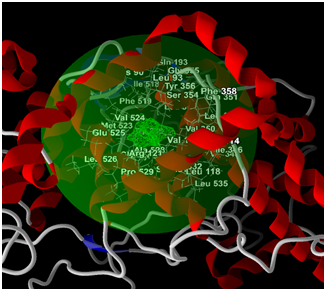


Fig SF6: Predicted binding cavity of the COX-2 (PDB ID: 4PH9)


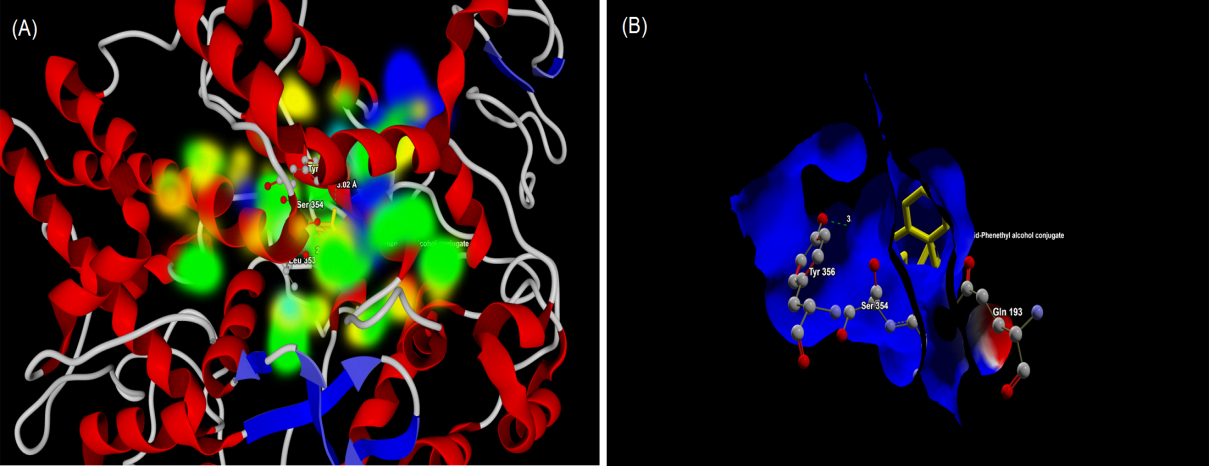


Fig SF7: (A) Energy map of COX-2 interacting with SAPE depicting steric interaction favourable (green), hydrogen acceptor favourable (turquoise colour), hydrogen donor favourable (yellow colour) and electrostatic favourable (blue and red colour) regions. (B) Electrostatic interactionof SAPE at the enzyme active site indicating electronegative (blue) and electropositive regions (red)


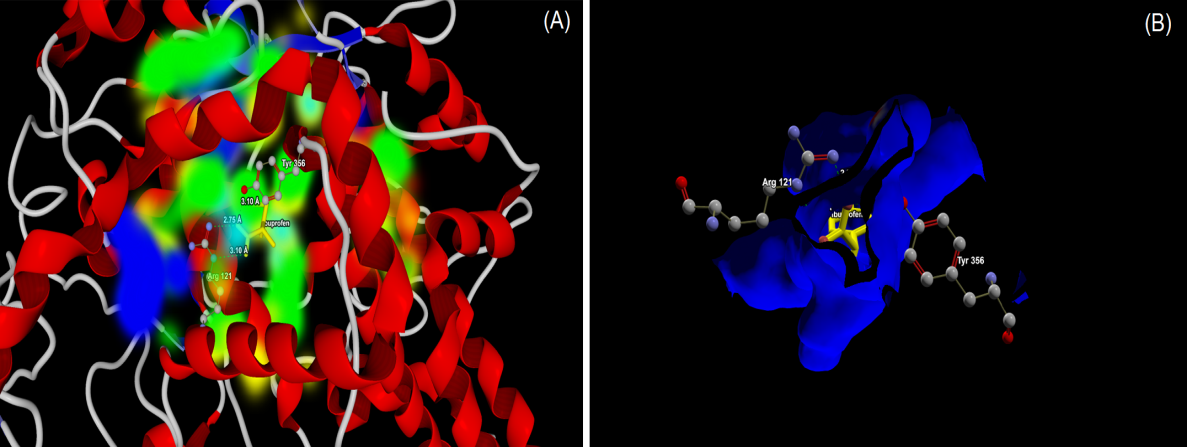


Fig SF8: (A) Energy map of COX-2 interacting with Ibuprofen depicting steric interaction favourable (green), hydrogen acceptor favourable (turquoise colour), hydrogen donor favourable (yellow colour) and electrostatic favourable (blue and red colour) regions. (B) Electrostatic interactionof Ibuprofen at the enzyme active site indicating electronegative (blue) and electropositive.


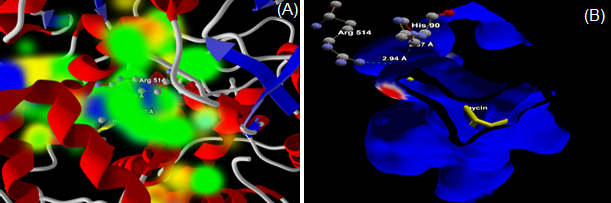


Fig SF9: (A) Energy map of COX-2 interacting with indomethacin depicting steric interaction favourable (green), hydrogen acceptor favourable (turquoise colour), hydrogen donor favourable (yellow colour) and electrostatic favourable (blue and red colour) regions. (B) Electrostatic interaction of indomehtacin at the enzyme active site indicating electronegative (blue) and electropositive.


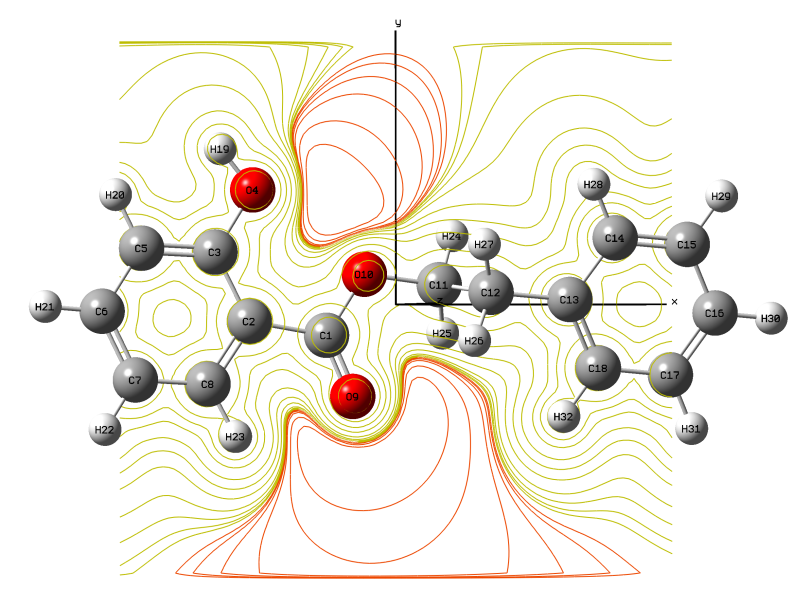


Fig SF10. Contour map depicting the electrostatic potential of SAPE calculated at DFT/B3LYP/6-31G level of theory.


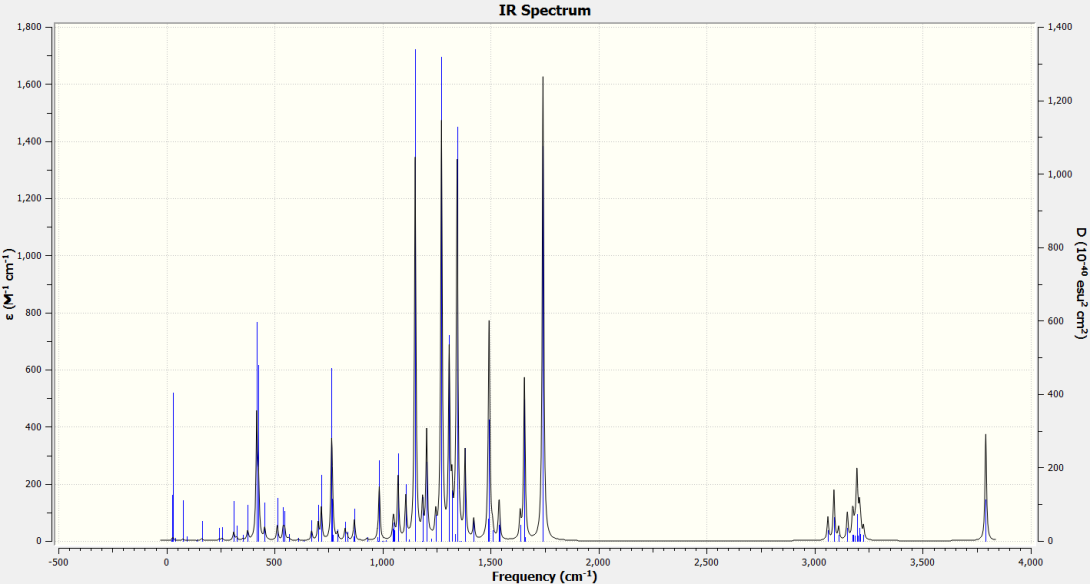


Fig SF11. Predicted IR Spectrum of SAPE calculated at DFT/B3LYP/6-31G level of theory.


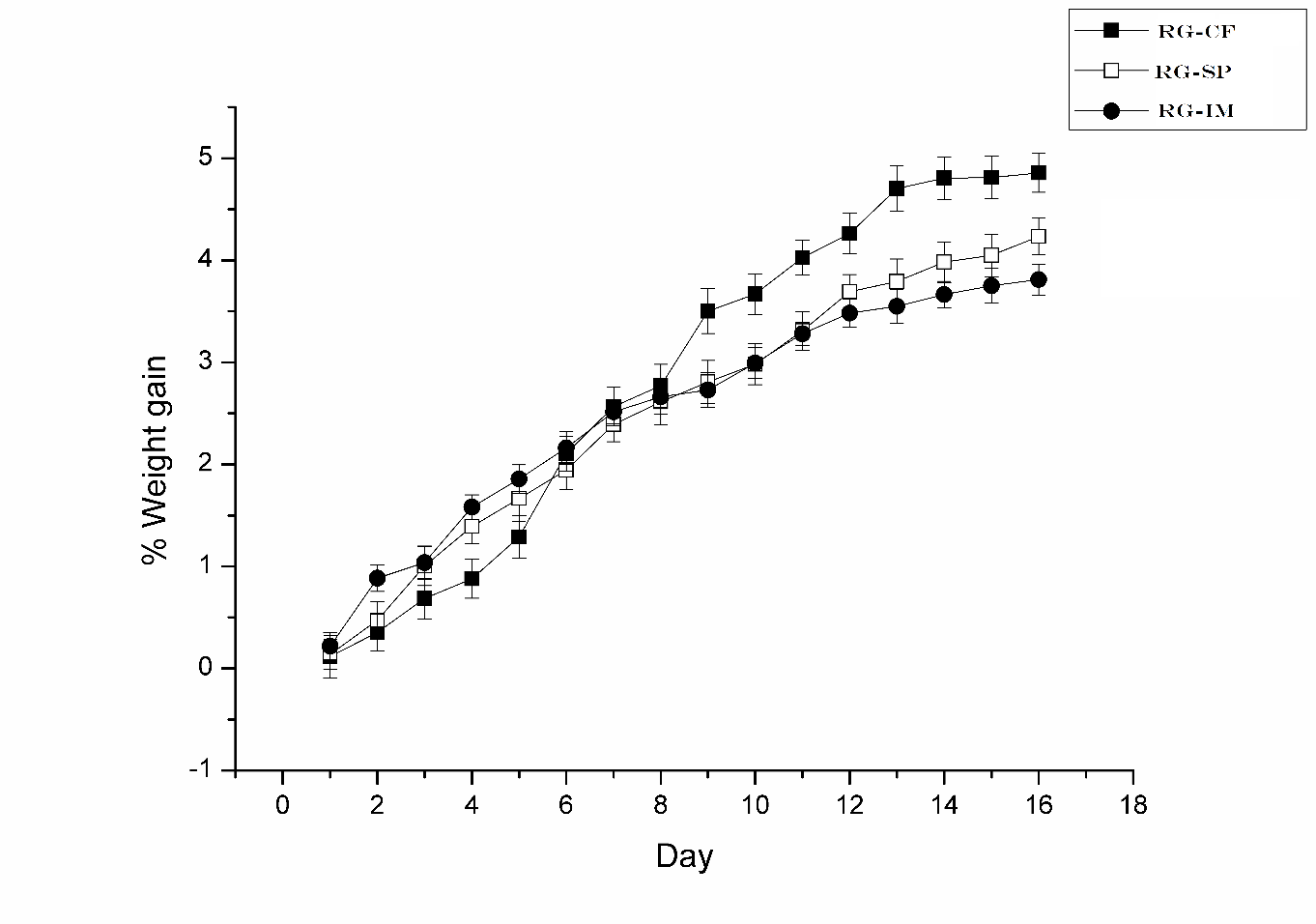


Fig SF12: Change in body weight of the different groups of rats (RG-CF: control without any treatment; RG-SP: treated with SAPE; RG-IM: treated with indomethacin) until the day of induction of colitis

**Tables:**

Table ST1: Absorption, distribution, metabolism, and excretion (ADME) studies related to SAPE

| Parameters | Activity of SAPE | | |
| --- | --- | --- | --- |
| Solubility | LogSw (AB/LogSw 2.0): -4.03  Reliability: Moderate (RI = 0.69)  Sw: 0.022 mg/ml | | |
| Volume of distribution | Vd: 0.44 L/kg  Acid (acid pKa<7.5 and no basic groups with pKa>6). Drugs in thisgroup have small Vd values (95% of these values are less than 1L/kg). | | |
| Absorption | Main physico-chemical determinants | LogP: 4.15  pKa (Acid): 3.70  pKa (Base): No pKa | |
|  | Maximum passive absorption: 100% | Contribution from:  Trancellular route = 100%  Paracellular route = 0% | |
|  | Permeability | Human jejunum scale (pH=6.5):  Pe, Jejunum = 7.51x10^-4^ cm/s | |
|  | Absorption rate | Ka = 0.051 min^-1^ | |
| Blood brain barrier transport | Main physico-chemical determinants | LogP: 4.15  pKa (Acid):3.7  Fraction unbound in plasma: 0.0335  pKa (Base): No pKa | |
|  | BBB transport parameters | Rate of brain penetration:LogPS: -1.8  Extent of brain penetration:LogPB: -0.2  Brain/plasma equilibration rate:Log(PS*fu, brain): -3.1 | |
| Bioavailability | Oral bioavailability between 30% and 70% | Probability that compound has:  %F(Oral) > 30%: 0.811  %F(Oral) > 70%: 0.358 | |
|  | Positive for | Solubility, Stability (pH < 2), Passive absorption, First-pass metabolism, P-gp efflux | |
| Health effects | Probability of effect | Blood: 0.39; Cardiovascular system: 0.5; Gastrointestinal system: 0.6; Kidney:0.26  Liver: 0.14; Lungs: 0.19 | |
| LD_50_ values | Species/Administration | LD50 (mg/kg) | Reliability (RI) |
|  | Mouse/Intraperitoneal | 610 | Moderate(0.65) |
|  | Mouse/Oral | 2100 | High(0.82) |
|  | Mouse/Intravenous | 85 | Borderline(0.39) |
|  | Mouse/Subcutaneous | 380 | Moderate(0.68) |
|  | Rat/Intraperitoneal | 570 | Moderate(0.54) |
|  | Rat/Oral | 2500 | Borderline(0.43) |

Table ST2: Physicochemical properties of SAPE

| Physical properties | | Lipinski-type properties | | Mass spectrometry related properties | |
| --- | --- | --- | --- | --- | --- |
| Molar refractivity | 71.85 ± 0.3 cm^3^ | Molecular weight | 240.3 | Monoisotopic mass | 240.11503 Da |
| Molar volume | 211.9 ± 3.0 cm^3^ | No. of hydrogen bond donors | 1 | Nominal mass | 240 Da |
| Parachor | 557.9 ± 4.0 cm^3^ | No. of hydrogen bond acceptors | 2 | Average mass | 240.297 Da |
| Index ofrefraction | 1.593 ± 0.02 | TPSA | 37.3 | M+ | 240.114481 Da |
| Surface tension | 47.9 ± 3.0 dyne/cm | No. of rotatable bonds | 5 | M- | 240.115578 Da |
| Density | 1.133 ± 0.06 g/cm^3^ |  |  | [M+H]+ | 241.122306 Da |
| Polarizability | 28.48 ± 0.5 10^-24^cm^3^ |  |  | [M+H]- | 241.123403 Da |
|  |  |  |  | [M-H]+ | 239.106656 Da |
|  |  |  |  | [M-H]- | 239.107753 Da |

Table ST3: Initial weight of the rats

| Group | Body weight (g) |
| --- | --- |
| RG-CI | 167.88±15.30 |
| RG-CF | 151.88±27.45 |
| RG-SP | 156.38±26.93 |
| RG-IM | 163.75±22.33 |
| RG-PO | 160.38±12.03 |

Note: Average weight of n=8±standard deviation (male =4; female =4)

Table ST4: Disease activity index (DAI) score table

| Score | Body weight loss (%) | Stool consistency | Faecal bleeding |
| --- | --- | --- | --- |
| 0 | 0 | Normal | Normal |
| 1 | 1-5 |  |  |
| 2 | 5-10 | Loose | Occult bleeding |
| 3 | 10-15 |  |  |
| 4 | >15 | Diarrhoea | Gross bleeding |

Note: Normal stool: shaped stool; Loose stool: pasty unformed stools which do not adhered to the anus; Diarrhoea: watery stools which adhered to the anus

Table ST5: Disease activity index scores of the different group of rats

|  | RG-CI | RG-CF | RG-SP | RG-IM |
| --- | --- | --- | --- | --- |
| DAI | 2.42 | 0 | 1.04 | 0.92 |

Note: Results are mean of 8 readings
